# A New Framework for Cortico-Striatal Plasticity: Behavioural Theory Meets In Vitro Data at the Reinforcement-Action Interface

**DOI:** 10.1371/journal.pbio.1002034

**Published:** 2015-01-06

**Authors:** Kevin N. Gurney, Mark D. Humphries, Peter Redgrave

**Affiliations:** 1 Department of Psychology, Adaptive Behaviour Research Group, University of Sheffield, United Kingdom; 2 INSIGNEO Institute for In Silico Medicine, University of Sheffield, United Kingdom; 3 Faculty of Life Sciences, University of Manchester, United Kingdom; UCL, United Kingdom

## Abstract

Operant learning requires that reinforcement signals interact with action representations at a suitable neural interface. Much evidence suggests that this occurs when phasic dopamine, acting as a reinforcement prediction error, gates plasticity at cortico-striatal synapses, and thereby changes the future likelihood of selecting the action(s) coded by striatal neurons. But this hypothesis faces serious challenges. First, cortico-striatal plasticity is inexplicably complex, depending on spike timing, dopamine level, and dopamine receptor type. Second, there is a credit assignment problem—action selection signals occur long before the consequent dopamine reinforcement signal. Third, the two types of striatal output neuron have apparently opposite effects on action selection. Whether these factors rule out the interface hypothesis and how they interact to produce reinforcement learning is unknown. We present a computational framework that addresses these challenges. We first predict the expected activity changes over an operant task for both types of action-coding striatal neuron, and show they co-operate to promote action selection in learning and compete to promote action suppression in extinction. Separately, we derive a complete model of dopamine and spike-timing dependent cortico-striatal plasticity from in vitro data. We then show this model produces the predicted activity changes necessary for learning and extinction in an operant task, a remarkable convergence of a bottom-up data-driven plasticity model with the top-down behavioural requirements of learning theory. Moreover, we show the complex dependencies of cortico-striatal plasticity are not only sufficient but necessary for learning and extinction. Validating the model, we show it can account for behavioural data describing extinction, renewal, and reacquisition, and replicate in vitro experimental data on cortico-striatal plasticity. By bridging the levels between the single synapse and behaviour, our model shows how striatum acts as the action-reinforcement interface.

## Abstract 2

A computational model yields new insights into the bewildering complexity of cortico-striatal plasticity and its rationale for supporting operant learning.

## Author Summary

A key component of survival is the ability to learn which actions, in what contexts, yield useful and rewarding outcomes. Actions are encoded in the brain in the cortex but, as many actions are possible at any one time, there needs to be a mechanism to select which one is to be performed. This problem of action selection is mediated by a set of nuclei known as the basal ganglia, which receive convergent “action requests” from all over the cortex and select the one that is currently most important. Working out which is most important is determined by the strength of the input from each action request: the stronger the connection, the more important that action. Understanding learning thus requires understanding how that strength is changed by the outcome of each action. We built a computational model that demonstrates how the brain's internal signal for outcome (carried by the neurotransmitter dopamine) changes the strength of these cortical connections to learn the selection of rewarded actions, and the suppression of unrewarded ones. Our model shows how several known signals in the brain work together to shape the influence of cortical inputs to the basal ganglia at the interface between our actions and their outcomes.

## Introduction

Learning from reinforcement requires a neural interface between reinforcement signals and action representations. Since the tentative identification of the ventral striatum as this “limbic-motor” interface by Mogenson and colleagues [1], separate strands of work have elaborated four key elements centred on the striatum. First, that phasic activity of midbrain dopamine neurons signals a prediction error between expected and received reinforcement, or the stimuli that predict reinforcement [2]–[5]. Second, that in the primary target for these signals, the striatum, the plasticity of cortical inputs to striatal medium spiny neurons (MSNs) is modulated by dopamine [6]–[8]. Third, that intact regions of striatum are necessary for the expression and likely acquisition of goal-directed and habitual actions [9]–[11]. Fourth, that the basal ganglia, for which the striatum is the input station, collectively implement a system for action selection via selective disinhibition of targets in motor thalamus and brainstem [12]–[14]. Consequently, a plausible hypothesis for the reinforcement-action interface is the interaction between cortico-striatal weights and phasic dopamine. Thus, the adjustment of cortico-striatal weights by value-conditioned environmental feedback, in the form of the phasic dopamine signal, changes which actions are prioritised in future [15].

Despite the extent of work on each of these elements, to our knowledge no model has integrated them all to test this widely held hypothesis. Such a model is required to tackle three critical challenges to this hypothesis. First, theories of reinforcement learning by the basal ganglia are based on simple dichotomies for cortical-striatal plasticity: that low and high dopamine respectively promote long-term depression (LTD) and long-term potentiation (LTP) at cortico-striatal synapses [15]; or in a more nuanced version that high dopamine promotes LTP at cortical synapses on D1-receptor expressing MSNs and low dopamine levels promote LTP at cortical synapses on D2-receptor expressing MSNs [16]. However, a recent study by Shen and colleagues [17] showed that whether these synapses express LTP or LTD is dependent on a three-way interaction between pre- and postsynaptic spike timing, postsynaptic dopamine receptor type (D1 versus D2 expressing MSNs) and dopamine level. Moreover, no combination of these factors maps onto a simple dichotomy. It is thus an open question whether this complex combination of plasticity rules can be reconciled with the reinforcement learning hypothesis.

Second, the D1 and D2 MSN populations project through separate pathways that converge in the output nuclei of basal ganglia. A broad class of hypotheses propose that these “direct” and “indirect” pathways respectively permit and prevent the selection of specific actions [16],[18]–[20]. It is unclear whether the just-described different plasticity rules operating on the cortical inputs to these pathways can be reconciled with this functional hypothesis.

Third, the timing of the relevant signals spans many scales. At short time scales (∼10–100 ms) cortical synapses onto the MSNs have spike-timing dependent plasticity (STDP) [21],[22]. At longer time scales (hundreds of milliseconds to greater than 1 s), there is the well-known credit assignment problem [23],[24]: that cortical-striatal signals for action selection appear transiently, and long before the phasic dopamine signal carrying feedback from the environment arrives in the striatum [4]. How the short-term STDP and long-term feedback interact is unknown.

We present here a model that provides the basis for integrating these strands of work on reinforcement learning and answering these challenges. It bridges the gap between the intricate subtleties of cortico-striatal plasticity at the synaptic level and the behaviour of the whole animal, thereby providing strong evidence that the striatum is indeed the locus of the action-reinforcement interface.

## Results

Our goal here is to explain how the complexities of dopamine-dependent cortico-striatal plasticity can ultimately give rise to the behavioural learning and suppression of actions driven solely by environmental feedback. The common point of reference is thus the MSN: how the combined effects of many cortico-striatal synapses on one neuron give rise to its changes in activity over learning, and in turn how the changed activity of a population of MSNs gives rise to changes in behaviour.

We first derive predictions for changes in D1 and D2 MSN activity over learning and extinction, by finding the required MSN activity for successful action selection or suppression in a network model of the whole basal ganglia that is consistent with recent electrophysiological studies on the D1 and D2 MSN pathways [19],[20],[25]. We then derive a three-factor cortico-striatal plasticity model for a single synapse from the in vitro data of Shen and colleagues [17], and extend to incorporate arbitrary levels of dopamine and an eligibility trace. The action selection and plasticity models are thus entirely independent of each other. The key test occurs when we link the two: can our in vitro derived plasticity rules at single synapses give rise to the predicted changes in MSN activity in both D1 and D2 pathways necessary for successful learning by reinforcement and extinction of a single action?

### Bridging the Gap between Plasticity and Behaviour

**Figure 1:**
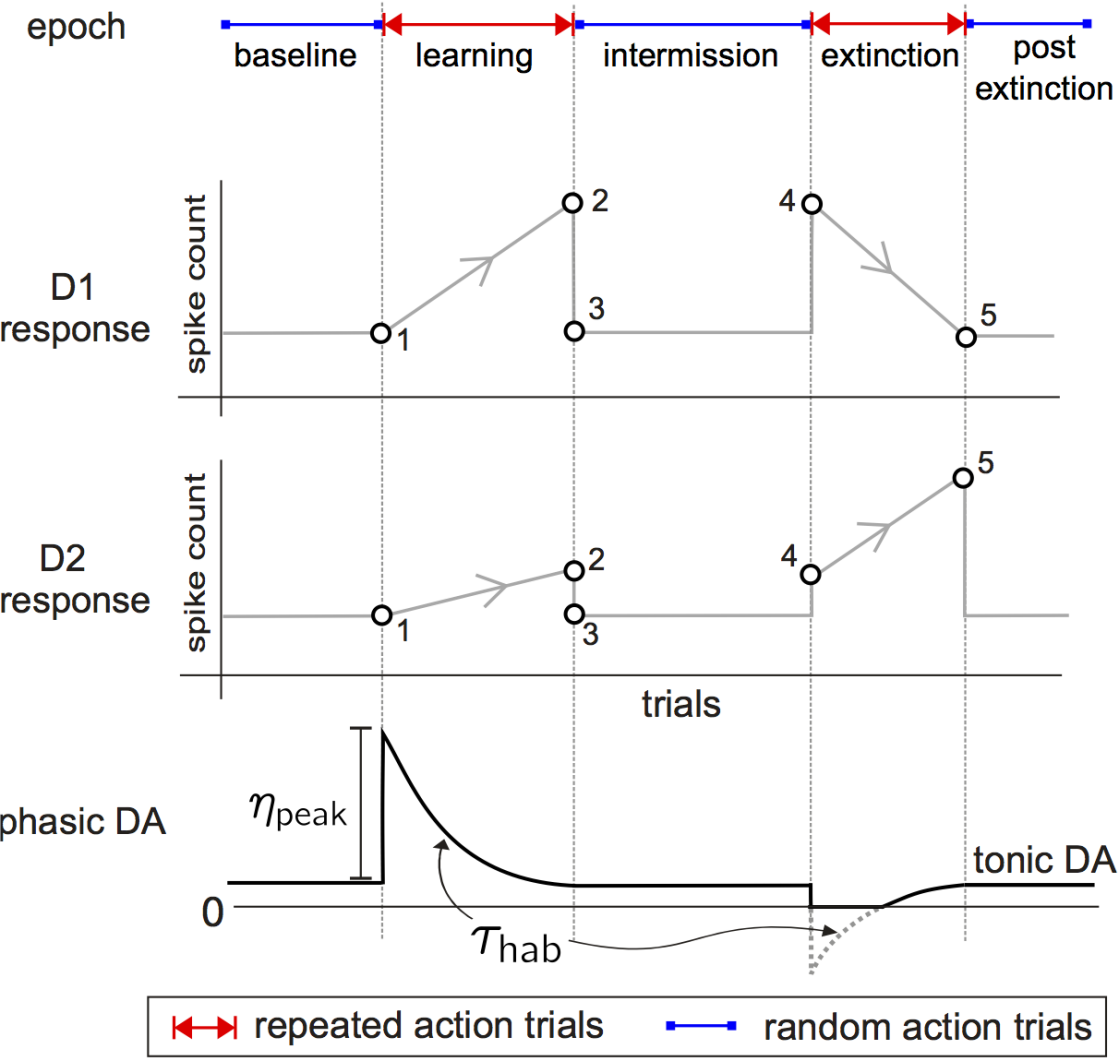
Stylised behavioural experiment for action discovery, with associated dynamics of MSN responses and phasic dopamine. The timeline at the top shows the experiment’s epochs. Below that we plot target response profiles of D1 and D2 type MSNs over each epoch of trials. These are based on the analysis in Figure 3 with the key points from that analysis shown by open symbols; grey lines between them show direction of change over the epoch. Stability is indicated by horizontal lines, and continuous (but not necessarily linear) plastic change is shown by lines with arrows between two open symbols. Bottom plot: trial-by-trial envelope-of-amplitudes of individual phasic dopamine events within each trial. This amplitude is governed by a variable 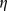, whose value decays exponentially when describing positive dopamine signals (bursts) from some maximal value 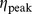. For negative going dopamine signals (dips) 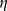 rises exponentially over a trajectory that can be negative (dotted grey line). However, the phasic excursions of the level of dopamine itself, 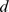, are always positive or zero, for when 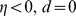. The use of 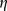 in this way expediently fixes the interval over which 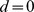. In both cases the time constant of the dynamics of 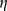 is 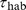.

To ground this exercise we imagine a stylised instrumental conditioning experiment with reinforcement learning of an action, such as a rat lever pressing for food pellet (in the Discussion we consider how our model of this task and of the inputs to striatum relate to the well-known distinction between goal-directed and habitual behaviour in instrumental tasks). We separate the experiment into epochs, and divide each epoch into notional trials corresponding to one action and its outcome. The timeline for the experiment is shown at the top of Figure 1.

Initially, there is a “baseline” epoch of free action choice. Following this, there is a “learning” epoch in which a key action–such as a lever press–is reliably paired with reinforcement, and consequently repeated. In the subsequent “intermission” epoch, the rat is removed from the arena and again has free action choice. This is followed by an “extinction” epoch, where the rat is reintroduced into the arena, but reinforcement is no longer paired with the previously reinforced action. We assume there ensues a period of repeated (but unsuccessful) attempts to obtain reinforcement. At some point the animal extinguishes its reinforced action and engages in a final bout of free-choice action in the “post-extinction” epoch. The baseline and intermission epochs will serve as controls for the models, testing that the absence of reinforcement does not lead to aberrant learning through noise (in baseline) and that the execution of other actions does not interfere with the learnt representation of the reinforced action (in intermission).

There is considerable in vivo evidence that striatal activity evolves during the course of operant learning, with both increases and decreases in activity observed, consistent with the hypothesis of cortico-striatal plasticity driving changes in activity over learning [26]–[31]. However, detailed interpretation of these data is difficult as there is no distinction made between D1- and D2-type MSNs. By contrast there are good recent data on the opposing roles of D1 and D2 MSNs in controlling behaviour, from which we can establish predictions for the start and end-points of learning and extinction. Cui and colleagues [25] showed that the execution of a specific action was immediately preceded by coincident activation of both D1 and D2 MSNs, showing that both direct and indirect pathways are active when selecting an action. Selective optogenetic stimulation has shown that activating D1 MSNs initiates or increases locomotion whereas activating D2 MSNs ceases or prevents locomotion [19],[20],[32].

Together, these data support the broad hypothesis for the competing influence of the two pathways on action selection, that D1 MSN activity is permissive for action and D2 MSN activity is preventative for action [18]. In the context of learning, this hypothesis has been interpreted as the D1 and D2 MSNs, respectively, learning the go and no-go contexts for a given action [16]. Optogenetic stimulation during learning suggests this interpretation is correct [33]. We here hypothesise that this extends beyond active suppression of an action in a specific context (no-go learning) to also include active suppression of a learnt action in extinction—we later show this hypothesis is consistent with renewal and reacquisition phenomena.

Currently missing are data or hypotheses for how the representation of the same action in corresponding D1 and D2 MSN populations changes over learning and over extinction. A straightforward extension of the competing pathways hypothesis is that after learning D1 MSN activity will be high and corresponding D2 MSN activity will be low or zero, thus favouring the selection of the action; and conversely that after extinction D1 MSN activity will be low or zero and D2 MSN activity high, thus favouring the suppression of the action. We used our prior model of action selection in the basal ganglia [34],[35] to test this hypothesis and predict the relative responsiveness of D1 and D2 MSNs that optimises selection performance within a trial after learning or after subsequent extinction.

### Relative Responsiveness of D1 and D2 MSNs for Optimised Action Selection

**Figure 2:**
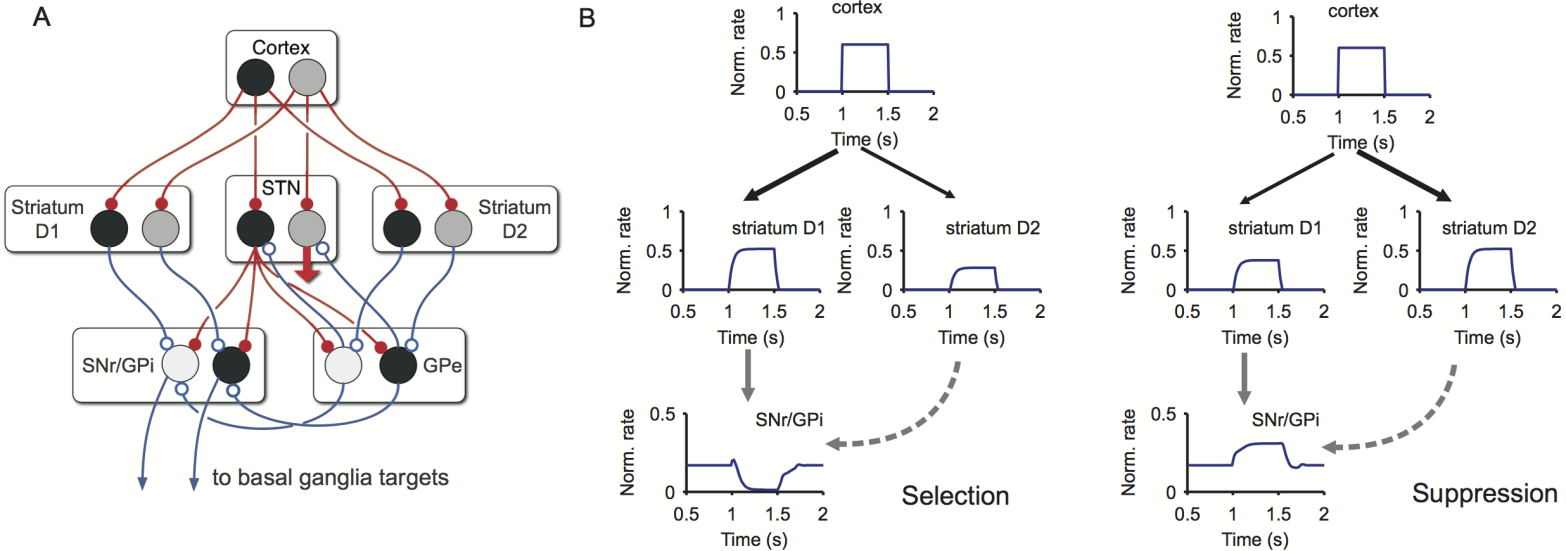
Model of basal ganglia dynamics. (A) Schematic of model architecture. It contains all major nuclei: STN, subthalamic nucleus; GPe, globus pallidus external segment; output nuclei (collectively)— SNr, substantia nigra pars compacta, and GPi, globus pallidus internal segment; striatum, with MSNs preferentially expressing D1 and D2 type dopamine receptors. Red and blue lines indicate excitatory and inhibitory connections, respectively. Circles indicate action-representing populations within each nucleus, each population modelled by its normalised mean firing rate, with relative rates represented by degree of shading (dark is highly active, pale grey is less so). In the interests of clarity, only two of the six channels are shown, and the diffuse projection from the channel on the right hand side in STN is shown as a single, wide red arrow (but mirrors its left-hand counterpart in terms of its individual connections to SNr/GPi and GPe). (B) Selection (left) and suppression (right) in the dynamical model. A phasic signal from cortex is input to a single channel in the model. Left: If the cortico-striatal weight is stronger to the channel’s D1 MSN population, then selection results: the corresponding SNr/GPi population’s activity is inhibited. Right: if the cortico-striatal weight is stronger to the channel’s D2 MSN population, then suppression results: via the effect of the enhanced D2 MSN input to the STN-GPe loop, the corresponding SNr/GPi population’s activity is excited. The model thus shows that a single cortical input drives coincident activity in D1 and D2 MSN populations, and that even within a single action-representing channel the two pathways are antagonistic.

Our model of the basal ganglia simulates how their internal circuitry can resolve competition between salient inputs from cortex (Figure 2)—see Methods for a full description. Under the interpretation that basal ganglia mediate action selection [12]–[14], cortical signals afferent to striatum associated with a single potential action comprise an “action request” [36]. The neural populations throughout basal ganglia that process this request comprise an action “channel.” In general, an action request is a complex pattern of signals encoding the action whose overall level of activity represents the “salience” or urgency of the request. Selection of an action is then signalled by a sufficient fall in the level of inhibition (relative to tonic) in the channel encoding the action in the basal ganglia’s output nuclei. Our model simulates the mean firing rate of each neural population within the basal ganglia in response to a given set of action requests.

Figure 2B shows the model’s response to a single phasic input from cortex. Consistent with the labelled-recording study of [25], a single action is represented by coincident activity in a small population of D1 and D2 MSNs. Consistent with the optogenetic stimulation studies of [19] and [20], activity in the two pathways is antagonistic: greater activity of the D1 MSN population drives inhibition of the corresponding basal ganglia output population, whereas greater activity of the D2 MSN population drives excitation of the corresponding basal ganglia output population. The model therefore shows that key to whether an action is selected or suppressed is the relative weighting of cortical input to the D1 and D2 MSN populations representing that action.

We thus used our model to find the relative weights of cortical input to the D1 and D2 MSN populations that optimised selection of an action (emulating the target situation at the end of the learning epoch) and, separately, that optimised the suppression of an action (emulating the target situation at the end of the extinction epoch). The ability to select a particular action can only be tested with reference to at least one other possible alternative action, so we considered two competing signals, one signal representing a fixed “control” action, available for selection throughout, and another signal representing the key action learnt and extinguished over the course of the experiment. We input this pair of salient signals to two channels in the model. For a given pair of inputs, we read out the outcome of the competition from the output of the basal ganglia (SNr/GPi in Figure 2): a sufficient decrease in inhibition from the output population signalled selection of the corresponding action. Thus three outcomes were possible: no action selected, one action selected, or both actions selected.

**Figure 3:**
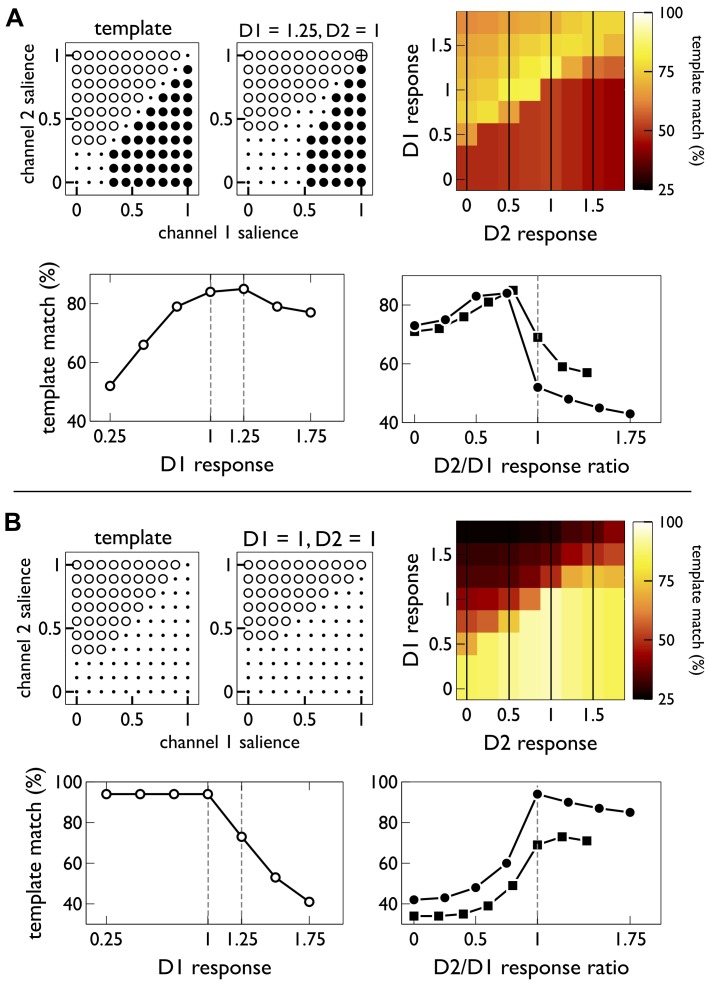
Linking action selection in basal ganglia to MSN responses. In all plots, neural “responsiveness” is the ratio of the population′s input value to output response; we abbreviate to “response” in axis labelling for brevity. (A and B) relate to action learning and extinction, respectively. The pairs of “bubble plots” in the top left of each panel show (i) an idealised selection template for a two-channel competition (left plot in each pair), with the key action on channel 1 and the control action on channel 2; and (ii) the best match to that template (at the D1 and D2 responsiveness noted above the plot). In each bubble plot, open symbols show an outcome of channel 2 selected, closed symbols show channel 1 selected, dots are no selection, and the crossed-circle shows both channels selected. The 2D colour plots (“heat maps”) show the template match for each D1/D2 responsiveness pair. The pairs of line plots show details of the corresponding colour map. The left hand line plots (open symbols) show the maximum template match for a given D1-MSN responsiveness; results at 1 and 1.25 are highlighted by the dashed lines. The right hand line plots (closed symbols) show cross sections through the 2D heat map (indicated by dashed grey lines therein) at D1 responsiveness of 1 (circles) and 1.25 (squares).

Given these possible outcomes for each input pair, we defined ideal outcomes for a range of pairs of salience values, shown at the top left of Figure 3A and 3B for selection and suppression, respectively. We expect low salience signals to give no selection as the unresponsiveness of MSNs to low inputs ensures that these signals do not change basal ganglia output [34]. Otherwise, for selection we expect the input with the highest salience to win and thus a single action to be selected; and for suppression we expect no selection of the suppressed action, and only selection of the control action when it is sufficiently salient.

Figure 3A shows that selection of an action was best achieved when its coding D1 MSN population was more responsive than its coding D2 MSN population. But, importantly, our results show that the best selection was achieved with some activity in the action’s coding D2 MSN population (Figure 3A, bottom right), suggesting the novel prediction that D2 MSN activity must also be present to achieve optimal selection, and so does not only block selection (in Figure S1 and Text S1, we explain why the model makes this prediction).

Figure 3B shows that suppression of an action was best achieved when its coding D2 MSN population was more responsive than its coding D1 MSN population. Importantly, our results showed that the action-coding D1 MSN population could remain highly active, with an lower limit of about 1∶1 for its input to output ratio. These results show that, rather than requiring that the D1 MSN input weight falls close to zero, the suppression of an action is robust to a large range of such weights.

### Resultant Hypotheses for MSN Activity Changes over Learning

Our model thus shows that the competing-pathways hypothesis is broadly true for the D1 and D2 populations coding a single action, but more nuanced: there is a non-intuitive contribution of D2 MSN activity to optimal selection; and successful suppression can tolerate high levels of D1 MSN activity. We capture these non-intuitive predictions as the hypothesized target activity at end-points of learning and extinction during the stylised experiment in Figure 1 (respectively, symbols 2 and 5).

There, we extend these end-points to their changes over the entire experiment with mild assumptions for MSN activity outside periods of learning. In the baseline epoch we assume a small, but non-zero response in both D1- and D2-MSNs, which is sufficient to initiate learning. In addition we demand that this baseline response is relatively stable during this period, such that randomly occurring pre- and postsynaptic spike pairings in this baseline activity do not cause either LTP or LTD. For similar reasons, we require stable responses in the intermission and post-extinction epochs. These profiles form the predicted targets for changes in MSN activity over learning for the rest of the paper.

The key hypothesis is that these changes in MSN activity are driven by feedback from changes in the environment that are carried by dopamine signalling in the striatum. The bottom panel of Figure 1 plots the corresponding trial-by-trial change in striatal dopamine during the behavioural task. Throughout the baseline, intermission, and post-extinction epochs, the absence of any reinforcing stimuli is reflected in the constant tonic dopamine level on every trial. At the onset of the learning epoch, the initial reinforcement, being unexpected, is assumed to elicit a phasic dopamine burst [2]–[4],[37],[38]. As the reinforcement becomes predictable, the amplitude of elicited phasic dopamine declines [39]. During the extinction epoch, the omission of the expected reinforcement is assumed to elicit phasic dopamine “dips” [2],[37],[38],[40], whose magnitude gradually declines, as the omission too becomes predictable [41].

### New Framework for Cortico-Striatal Plasticity

With these target trial-by-trial changes in MSN activity and corresponding striatal dopamine profile in hand we turn to the central question of how that dopamine signal drives the required MSN activity changes. The long-standing answer has been that dopamine modulates cortico-striatal plasticity [15], but recent data have shown a partially complete picture of how nuanced that modulation is. On the one hand, Pawlak and Kerr [22] showed that cortico-striatal synapses have STDP, but not how that depends on postsynaptic neuron type (D1 or D2). On the other hand, Shen and colleagues [17] showed that the direction of modulation is dependent on the three factors of postsynaptic neuron type (D1 or D2), dopamine concentration (high or low), and the sign of pre- and postsynaptic event timing (positive or negative), but not how it depends on the delay itself.

We therefore used these data as the starting-point for a new framework for cortico-striatal plasticity. This framework extrapolates naturally from the data in three ways. First, it extrapolates from the Shen data to the STDP functions described by Pawlak and Kerr. Second, it establishes a simple way of defining plasticity rules over a continuum of dopamine levels, proposing dopamine-dependent STDP. Third, it incorporates an eligibility trace to solve the temporal credit assignment problem—that the change in dopamine level is locked to environmental feedback, and so occurs long after the signals for action are input at cortico-striatal synapses.

### From In Vitro Data to STDP Functions

**Figure 4:**
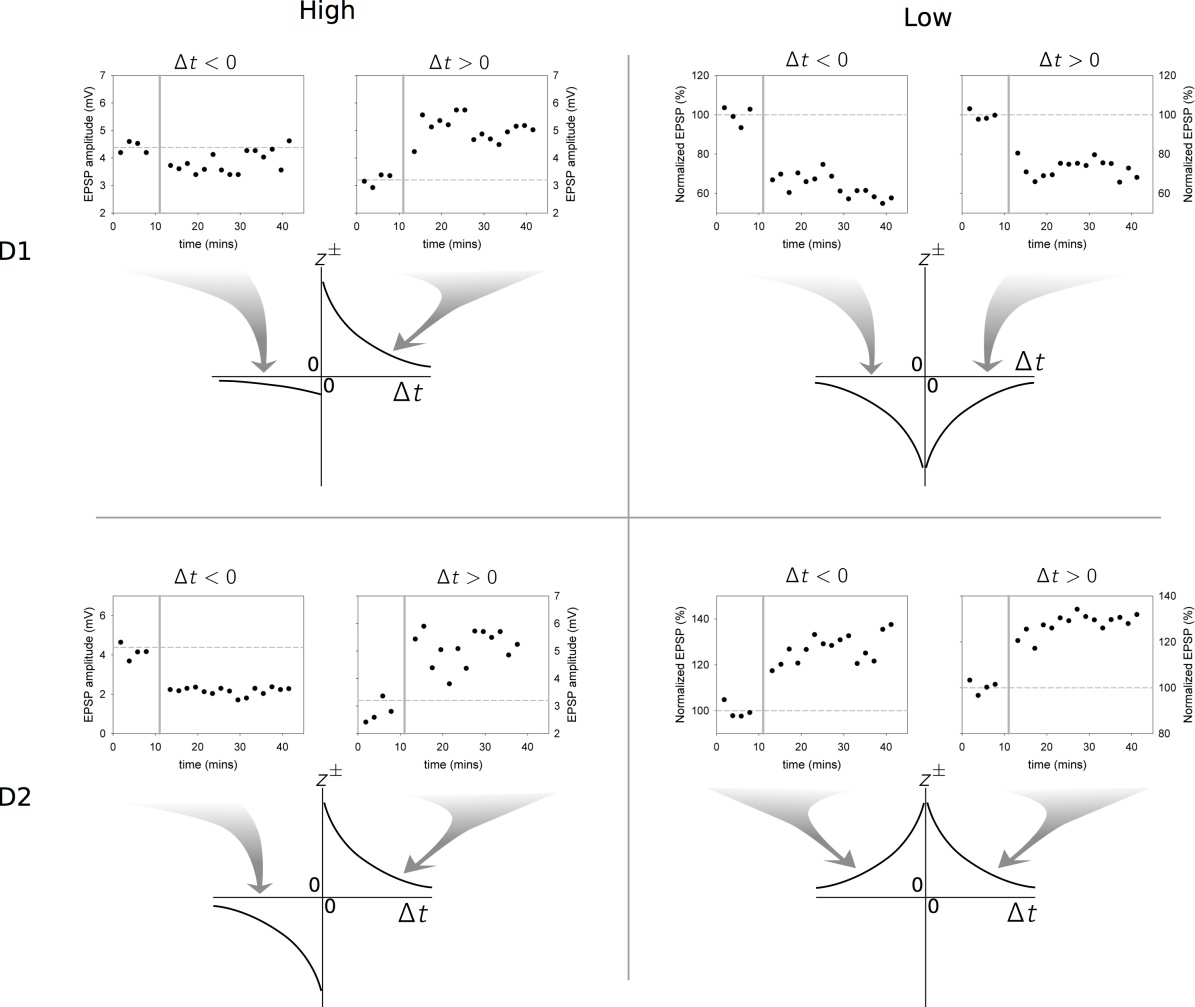
Deriving STDP functions from the in vitro data in [17]. Each row of the four panels pertains to an MSN type (D1, D2), each column to a dopamine level (“high” or dopamine present, and “low,” or dopamine depleted). Thus, the top left panel shows data for MSNs expressing D1 receptors from slices with dopamine present. In each panel, the top right hand plot shows the EPSP amplitude against time under protocols designed to induce Hebbian learning, and in which the postsynaptic spikes follow their pre-synaptic counterparts (“positive timing,” 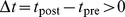). Plasticity induction occurred during the period indicated by the reference line at 10 mins. The top left hand plot in each panel shows corresponding results for negative timing, 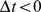. The resulting STDP functions (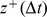 for 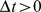 and 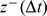 for 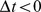) are shown in the cartoon diagrams, and the relation between data and function is indicated by the shaded arrows. Note that some of the vertical axes on the data plots show normalised EPSP amplitude as a percentage.

Figure 4 shows how we interpret the data of Shen and colleagues [17] in terms of STDP functions, generalising from the data of [22] by assuming that each combination of MSN type and sign of pre- and postsynaptic event timing has a standard exponential function of time [42].

The dopamine level 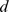 in the experiment is assigned one of two values—“high” or “low” (depleted)—where the term “high” is simply used as a contrast with “low” and no implication is made that this is a biologically high level. To deal with spike timing, let 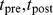 be a pair of presynaptic and postsynaptic spike times, respectively. Letting 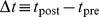, we refer to the conditions 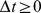, 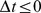 as “positive” and “negative” spike-pair timing, respectively. For a given pair of pre- and postsynaptic events separated by 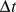, we model the exponential dependency of plasticity on timing by 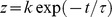, where 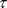 sets the time scale of the exponential decay, and coefficient 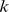 sets the scale of contribution to plasticity: high values of 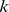 indicate a larger contribution. The consequent change in weight is 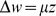, where 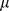 is a learning rate.

We define separate functions 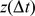 for each combination of receptor type (D1, D2), dopamine level (low, high), and sign of pre-post event timing (+, −) in the Shen and colleagues′ [17] data. As an example consider the case of low dopamine with D1-MSNs shown in the top right panel of Figure 4. For positive spike timing, the data show clear LTD and so we assign a negative function 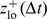 describing the relation between plasticity change and 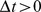, with amplitude 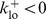 to capture the LTD in the data (note the “+” superscript refers to the positivity of 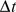, not the sign of the function value; “lo” indicates “low dopamine”). Duplicating this whole procedure for all other combinations results in a set of four plasticity coefficients for each of D1 and D2 type MSNs: 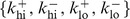.

Even at this qualitative stage of the model, our distillation of the complex dataset of Shen and colleagues [17] shows that their data imply “standard” STDP (LTP and LTD in positive and negative timing, respectively) applies only for D2 MSNs under high dopamine levels; all other combinations of MSN type and dopamine level imply non-standard combinations of LTP and LTD with pre- and postsynaptic spike timing.

### Extending the Model to Arbitrary Levels of Dopamine

**Figure 5:**
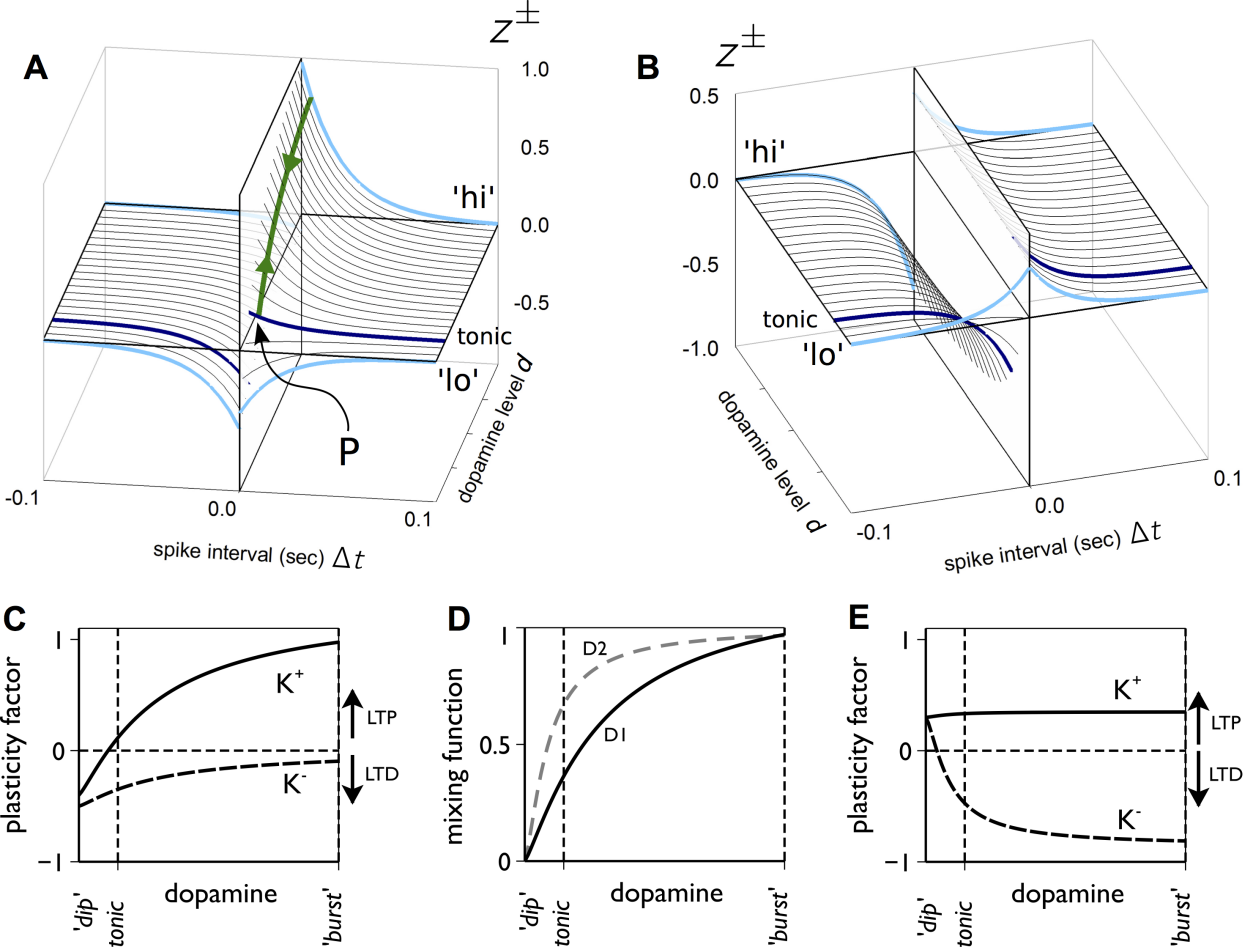
The “function mixing” model of dopamine-dependent cortico-striatal plasticity. The 3D plots in (A and B) are for D1 and D2 MSNs, respectively. In these plots, for constant levels of dopamine 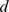, the thick, light-blue lines show the STDP functions 
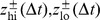
 at high and low dopamine levels corresponding to those in Figure 4. For other (constant) values of 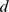, the STDP function 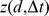 is obtained by smoothly “blending” together 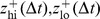 for positive timing, and 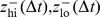 for negative timing. Thinner black lines show some examples of this, and the tonic dopamine level in our model gives functions shown in dark blue. With time-dependent levels of dopamine and eligibility, the generalised plasticity function 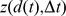 can change dynamically with time for a given 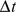. The green line in (A) shows a typical such trajectory as a phasic dopamine burst is received, starting at tonic level, moving to the peak of phasic amplitude and back again. (D) The mixing function 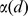 that determines how much of each of the functions, 
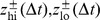
 are blended together across the range of dopamine 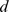. (C and E) The resultant plasticity factors for D1 (C) and D2 (E) MSNs, respectively, giving the amplitude of the STDP functions at 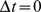 in (A) and (B).

In order to extend these results to arbitrary levels of dopamine 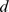, we define functions 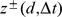 for any 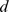 by smoothly mixing or “blending” the functions at the extremes of the range, 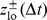 and 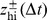, according to 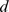: Figure 5D plots the particular mixing functions used here (see Methods). For a given level of dopamine, the mixing function determines the consequent amplitude 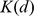 of the STDP functions, thus setting the change in weight—we plot these “plasticity factors” 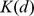 for each spike-timing (+, −) and receptor type (D1, D2) in Figure 5C (D1) and 5D (D2).

Figure 5A and 5B plots the resultant two-dimensional STDP functions over the full range of dopamine level 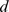 for D1 (Figure 5A) and D2 (Figure 5B) MSNs, showing that various combinations of LTP and LTD emerge naturally from the mixing scheme. In particular, the smooth morphing of the STDP functions predicts that, at some intermediate levels of dopamine, both D1 and D2 MSNs would express “standard” STDP; this case is highlighted by the dark blue lines in Figure 5A and 5B.

The parameters of the mixing function were chosen so that this standard STDP in both MSN types occurred at our nominal level of tonic dopamine. We expect such tonic dopamine to be present outside of the learning and extinction epochs (Figure 1), yet for there to be no change in synaptic strength despite the ongoing pre- and postsynaptic spike-pairings in background spiking activity. We show below that using these standard STDP functions at tonic dopamine levels indeed results in no overall change in synaptic strength outside learning and extinction.

### Extension to Longer Time Scales: Spike Timing Dependent Eligibility

In operant conditioning experiments schematised in Figure 1, at some time during or immediately after the action request, the action is executed, and any environmental consequences made apparent. If unpredicted, these will cause a phasic dopamine signal. The delay between action request and consequence is largely regulated by the physics of the world and can be as much as 1–2 s, or even longer, while still allowing action discovery [43]. There is therefore a temporal credit assignment problem [23],[24]: for if cortico-striatal plasticity is the proposed locus of reinforcement learning and is dopamine-dependent, how can the transient cortico-striatal action request lead to correct changes in cortico-striatal weights by dopamine signals arriving long afterwards?

Solutions often involve some kind of “eligibility trace” in which pre- and postsynaptic activity at a neuron establishes the potential for plasticity, which is later converted into permanent change with dopamine. Here we adopt the dopamine and STDP-dependent eligibility trace model introduced by Izhikevich [44], and extend by incorporating the non-standard forms of STDP and the plasticity-function mixing framework described above (see Methods for a formal description).

In this model, plasticity is not governed directly by the STDP functions; rather, these are used to establish an eligibility trace, which subsequently decays over time in the order of seconds. It is this trace, together with its interaction with dopamine, that governs synaptic weight change. We therefore refer to this plasticity framework as “spike timing dependent eligibility” (STDE).

**Figure 6:**
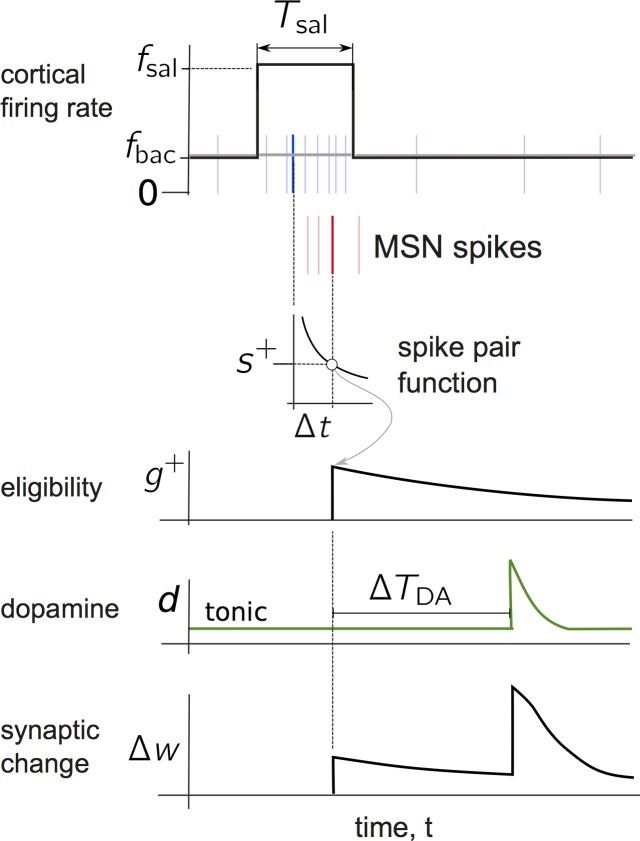
Cartoons of signals during a single trial of action learning. The top panel shows the firing rate of cortical neurons afferent to a particular MSN, and taking part in an action request. Afferent subset 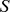, is distinguished by a higher firing rate 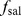 (solid black line) during the request, and its set complement, 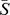, has afferents with rates at background levels 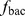 (grey line). The action request lasts for some period, 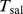, and outside this period, all afferents receive spikes at rate 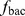. Typical cortical spikes are shown in blue with one highlighted in a darker hue. Just below these are shown a typical MSN response, (spikes in red, one highlighted in darker hue). The highlighted spike pair has an ISI of 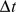 and elicits an contribution 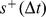 to the eligibility trace 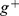 (promoting LTP in this case). The eligibility interacts with the dopamine signal to produce a contribution to the change 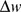 in synaptic strength. Notice that the phasic dopamine signal occurs at a time 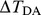 after the spike pair which is much longer than the time constant for the STDP function.

The process is illustrated for positive spike timing in Figure 6, which also shows our model of an action request—see below. Each pre- and postsynaptic spike pair for which 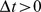 creates a step-change contribution 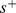 to an eligibility trace 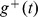, where 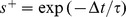 is the time dependent STDP function used previously. The eligibility decays exponentially with time constant 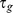, where 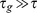, so the eligibility 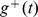, due to a single spike pair, is therefore 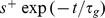.

In contrast to learning under STDP, STDE introduces time-dependence within a single trial of both dopamine level 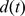
—describing the phasic dopamine response to environmental events (Figure 6, green trace)—and the eligibility trace 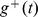. Thus each synaptic weight 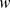 is updated continuously in STDE, with the change at time 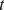 proportional to both the current state of the eligibility trace 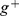 and the current dopamine level 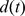, as shown in Figure 6. The magnitude of the change is still given by the dopamine-dependent plasticity factor 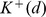, but now 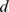 depends on time. Put together, the change in weight for positive spike-timing is thus proportional to 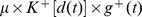.

The plasticity rule may be extended to spike pairs with negative timing by introducing an eligibility 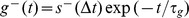. Overall plastic change at a single synapse is then the sum of contributions from both 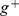 and 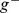. Multiple spike pairs are accommodated by assuming their contributions combine linearly. The learning rule was chosen so that, under constant dopamine, STDE reduces to STDP; that is, the overall change in synaptic strength for a spike pair is the same as that in STDP.

Later, we show that this STDE model of cortico-striatal plasticity is able to account for the original experimental data of Shen and colleagues [17]. Here, we continue with our programme relating plasticity to operant learning.

### STDE Plasticity Rules Produce Changes in Single MSN Activity Required for Operant Learning and Extinction

We now have on the one hand predicted D1 and D2 MSN activity changes over trials of an operant learning task, and on the other an in vitro-derived model for cortico-striatal synaptic plasticity as a function of given pre- and postsynaptic spike timing, MSN type, and dopamine level. Together these allowed us to test the basic hypothesis of reinforcement learning: that adjustment of cortico-striatal weights by value-conditioned environmental feedback, in the form of the phasic dopamine signal, changes which actions are prioritised in future.

To do so, we simulated the stylised experiment described above (Figure 1; see Methods for a formal description) using our previously developed spiking models of the D1 and D2-type MSNs [45] as representatives of the action-coding populations of D1 and D2 MSNs. The spiking model simulates background synaptic input from cortical (via AMPA and NMDA receptors) and intra-striatal (via GABA receptors) sources, and incorporates tonic dopamine modulation of the MSN’s excitability.

The top panel of Figure 6 shows the model of spiking input and dopamine feedback signals occurring around a single MSN during a single trial of the simulated experiment, comprising a single action and its possible reinforcement. Within each trial we simulate a phasic action request by a subset, 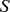, of cortical afferents to the MSN that generate a short burst of spikes with a higher firing rate than background levels, with the remaining afferent subset 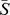 at background rate. Random action choice in the baseline and intermission epochs are modelled by randomly choosing the active subset of cortical signals, 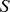, on each trial. During learning and extinction epochs, the same set 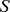 of cortical signals representing the reinforced action is transiently active in each trial of the epoch. Where reinforcement was presented (in learning) or expected (in extinction) the phasic dopamine signal on that trial was delayed by 150 ms. Across trials the magnitude of the dopamine signal changed according to the envelope shown in the bottom panel of Figure 1.

Each AMPA synapse of the model was updated using the STDE rules. Our only free parameters were thus the key plasticity coefficients 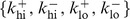, but these were constrained to have the correct sign for LTP or for LTD as shown in Figure 4 (that is, for D1 MSNs, 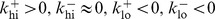 and for D2 MSNs 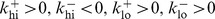).

**Figure 7:**
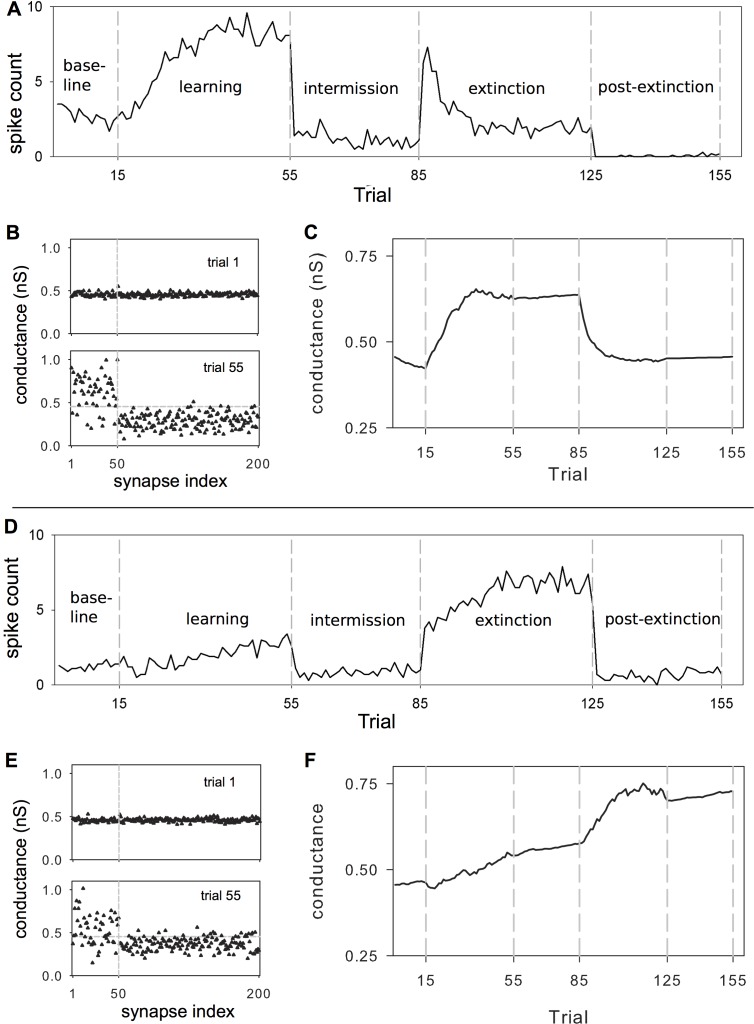
Behaviour of MSNs obtained with the STDE plasticity rules (we plot here means obtained over ten experiments with different random initialisation of AMPA conductances). (A) and (D) are the response profiles, for D1-MSNs and D2-MSNs, repectively, plotted as spike count per trial against trial number; key trials delimit epochs defined in Figure 1. Coefficients for D1-MSNs were: 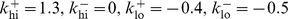; and for D2-MSNs were: 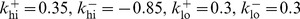. (B) and (E) the mean AMPA conductances for D1-, D2-MSNs of each synapse at trials 1 and 55. Synapses in the set, 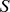, (50 synapses consistently subject to stronger input during the learning and extinction epochs), are collected at the left hand side of each plot, and delimited by the vertical dotted line. The horizontal dotted line shows the initial mean AMPA conductance of 0.458 nS. (C) and (F) The mean conductance of the set 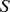 against trial number (D1-, D2-MSNs, respectively).

Within these constraints, we easily found coefficients that produced the target changes in activity for both D1 MSNs and D2 MSNs across all epochs of the simulated operant experiment. Figure 7A and 7D shows the resulting change in D1 and D2 MSN activity over the simulated experiment for an example well-performing set of coefficients. Thus, we see that dopamine-modulated STDE synapses can indeed drive the required activity changes in D1 and D2 MSNs despite reinforcement or its omission being delayed beyond the end of the STDP time-window.

We particularly note that the two unintuitive properties of the MSN responses derived from the network model arise naturally from the in vitro-derived STDE rules: first, that the reduction in D1-MSN activity over extinction need not drive this activity to zero, or even to the average activity of the preceding intermission epoch; second, that D2-MSN activity does increase during the learning epoch as a consequence of the STDE rules. In Figure S2 and Text S2 we further show that the resultant cortical input weights to the D1 and D2 MSN models from each epoch of the operant task do, in turn, produce the required action selection performance for the whole basal ganglia network model.

In both D1 and D2 MSN profiles, we also note there was no change in activity across trials in the baseline, intermission, or post-extinction epochs, showing that our choice of using the “standard” STDP functions at tonic dopamine levels (Figure 5) is indeed sufficient to suppress plastic change overall despite many pairs of pre- and postsynaptic spikes and the presence of dopamine.

These activity changes over the course of the experiment were driven by the dopamine-dependent changes in cortical input weights. We plot the evolution of the mean synaptic strengths (AMPA conductances) in the fixed afferent set 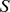 for D1-MSNs and D2-MSNs in Figure 7C and 7F, respectively; illustrative snapshots at trials 1 and 55 of the full synaptic sets are shown alongside in Figure 7B and 7E. There is clear evidence of the development of matching between the patterns of cortical signals and synaptic conductances in the fixed afferent set 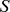. Note how, in both MSN types, conductances increase during the learning phase (compare outcome at key trials 1 and 55), and are preserved during free action choice of the intermission epoch (compare trials 55 and 85). For D1-MSNs the conductances in 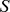 decrease during extinction, while for D2-MSNs they increase (compare across trials 55 and 125).

### Explaining Context-Dependent Renewal and Reacquisition

In constructing our target changes in MSN activity over learning we advanced the hypothesis that increased D2 MSN activity in extinction causes active suppression of a previously reinforced action. That this increased activity in extinction emerged from our STDE plasticity model (Figure 7D) is partial evidence in support of the hypothesis. To further test this hypothesis, we sought to determine whether the active suppression hypothesis could be reconciled with the post-extinction behavioural phenomenon of renewal (context-switch evoking immediate display of the previously acquired behaviour) and reacquisition of the key action (after a subsequent bout of reinforcement) [46]. Given that the action-representing weights for D1 MSNs returned to baseline after extinction (Figure 7C), while those for D2 MSNs reached their highest value (Figure 7F), it was not clear that the plasticity model could account for these post-extinction phenomena.

In renewal and reacquisition protocols, learning and extinction are carried out in two environments with differing contextual cues that may be visual, structural, or olfactory [47]. Typically an operant task is learned in a context 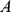, extinguished in context 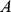, or another 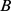, and behaviour then tested for renewal or reacquisition in a context different from that used during extinction. This leads to protocols 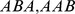, but results are also sometimes reported for control sequences 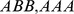, in which, unsurprisingly, the “renewal” performance is close to that observed at the end of extinction [48].

**Figure 8:**
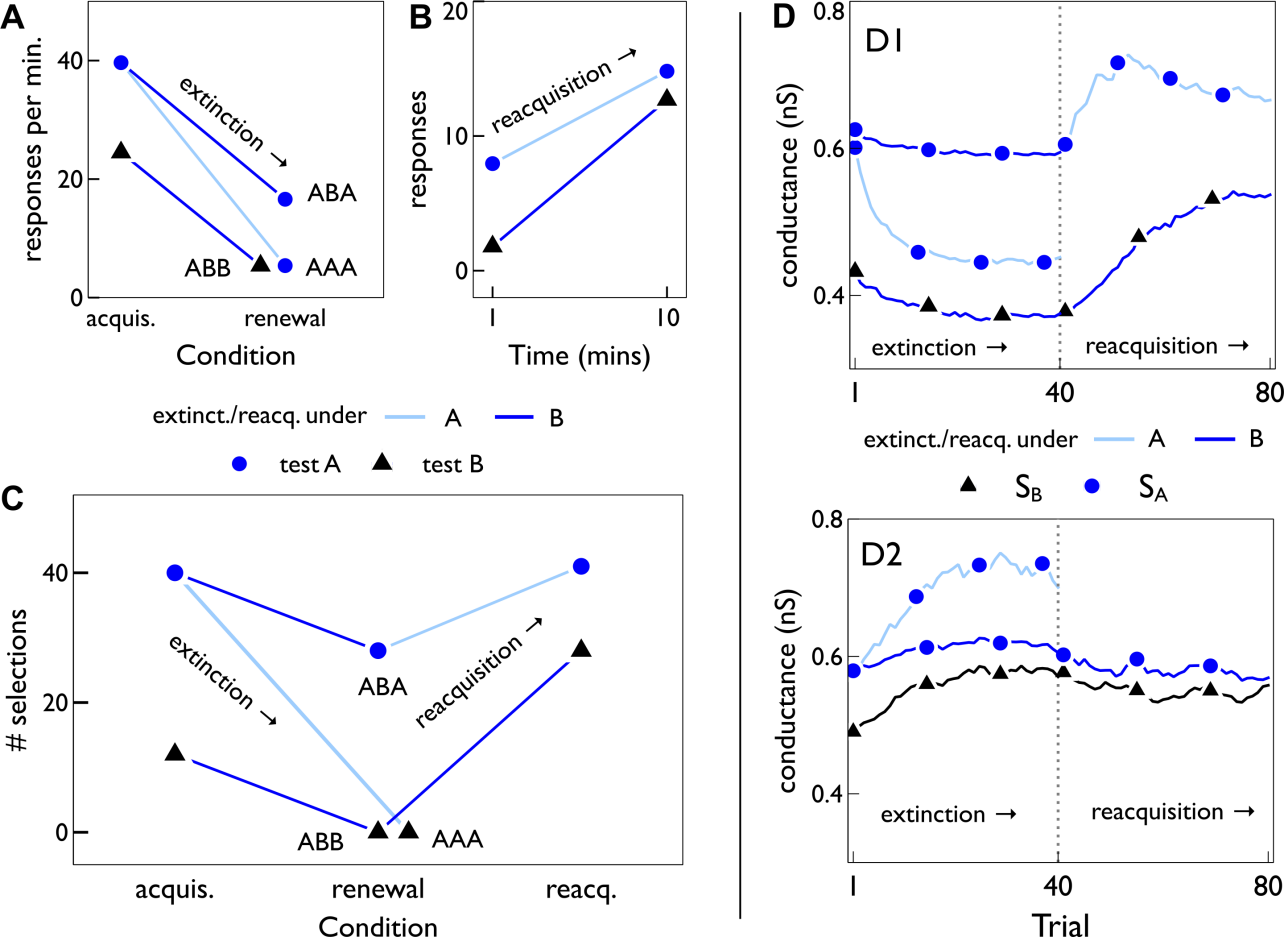
Extinction, renewal, and reacquisition. (A) Summary of relevant data from Figure 3 in [49], (see Methods for interpretation of that data) showing renewal effects after sequence *ABA*, but not after control sequences *AAA* and *ABB*. The points labelled “acquis.” are the performance before extinction in the same context as the renewal test, giving a baseline for the performance change caused only by any switch in context after extinction. In all of (A–C), the blue/black symbols correspond to testing with contexts 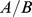. (B) Summary of relevant data from [50] (see Figure 2 therein), showing reacquisition of responding in two contexts 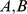, after original acquisition in 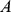 and extinction in 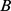. The symbols show endpoints of linear regressions through the original data, which include outcomes at several intermediate time points. (C) Behavioural responses of the basal ganglia model with MSNs initially trained with context 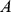. The acquisition (“acquis.”) is tested near the end of the intermission period for two contexts, 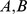, derived using different strong-afferent synaptic sets 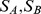 (see text for details). The renewal is tested at the end of 40 trials of extinction under both contexts, leading to the renewal sequences 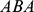, 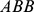, and 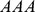. Reacquisition is measured after 40 learning trials, under each context. (D) Shows (for both D1- and D2-MSNs) the mean AMPA conductance of synaptic sets 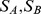 against trial number, during extinction (trials 1–40), and reacquisition (trials 41–80) under the behavioural protocols in (C). Trials are numbered from trial 80 near the end of the intermission period in the simulated experiment (Figure 7). The trajectory for 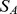 under extinction with 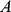 (pale blue line, dark blue symbols) is identical to the extinction shown in Figure 7.

Our goal was to test whether synaptic changes due to the STDE plasticity model could both allow renewal and cause reacquisition. To do so, we simulated these protocols using the spiking MSN model with STDE to find the changes in the cortico-striatal synaptic weights; to assess performance at the different stages of the protocols, we took the weights found at these stages and constructed equivalent rate-coded D1 and D2 MSNs, tested the resultant basal ganglia network model’s response behaviour, and compared it to experimental results. We did this for sequences 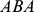 (test for renewal and reacquisition), 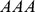 (control for the same context in learning and renewal/reacquisition), and 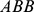 (control for the same context in extinction and renewal/reacquisition). Figure 8A shows a summary of relevant data from experiments by Nakajima and colleagues [49] (from their Figure 3) on extinction and renewal. We plot there the results of testing response behaviour in the context used for renewal both before extinction (point labelled ‘acquis.’—acquisition) as a control for the effect of changing the context alone, and after extinction (point labelled ‘renewal’). Figure 8B is a summary of relevant data from experiments in [50] on extinction and reacquisition (see Figure 2 therein)—see Methods for details of our interpretation.

In order to simulate the use of different contexts with the STDE-equipped MSN spiking model we manipulated the strongly active afferent synapse set 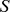. We assumed that 50% of the original set 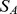, used to obtain the previous results, is responsible for sensory components common across contexts 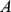 and 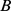, as well as any pre-motor components of the action request for the key action. We then established a new set 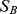, which included this 50% of 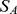, with the remaining half of its synapses drawn randomly from the set complement 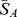. The cortical input under context 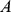 or 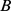 then takes the salient input value 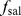 (see Figure 6) at synapses in 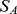 and 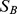, respectively. Using these input sets, we simulated the three sequences for the renewal protocol, and then tested for reacquisition in context 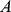 or 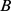 (reinstating the phasic dopamine signal in each trial to simulate the reintroduction of reinforcement).

The behavioural performance at each stage of the simulated sequences was determined by testing the response of the spiking D1 and D2 MSN models to cortical input at that stage (given their learnt weights), and using their responses to parameterise an equivalent rate-coded neuron that captures their learnt responsiveness at that stage of the sequence (see Methods). Embedding these in one channel representing the key action, the resultant basal ganglia network model was then tested with the paired-input protocol used to assess selection (Figure 3); the performance metric was the number of selections of the key action channel (channel 1), corresponding to the numbers of responses in the in vivo experiments.

Figure 8C shows that the model’s behavioural performance both before and after extinction is consistent with the data in Figure 8A: there is reduced selection of the key action under context 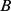 after initial acquisition, selection under renewal is always diminished with respect to corresponding acquisition performance, and selection under renewal in the 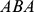 protocol is greater than that in the 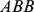 and 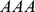 protocols. Figure 8C also shows that the model’s behavioural performance following the subsequent reintroduction of reinforcement is consistent with the data in Figure 8B: requisition allows increased selection, and the ordering under both contexts is preserved.

The relative cortico-striatal weight changes in contexts 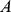 and 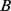 underpinned these performance changes. Figure 8D shows the trajectory of the mean AMPA conductance 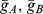 of each of the synaptic sets 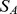, 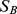, under learning with the protocols described above. As we might expect, at the start of extinction (Trial 1), 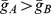, since learning has been carried out with respect to 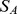. This accounts for the “acquisition” selection results in Figure 8C. In all cases, extinction causes a reduction/increase in mean conductance for D1/D2-MSNs, with both features promoting diminution of selection under “renewal.” However, the changes with extinction under context 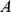 for synaptic set 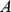 are most marked, which explains the correspondingly larger decrease in renewal selection under extinction with 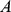. New learning under reacquisition causes increased/reduced conductances for D1/D2-MSNs resulting in the increased selection observed.

We thus found that active suppression of the key action by D2 MSNs during extinction could nonetheless give rise to its renewal and reacquisition.

### General Cortico-Striatal Plasticity Rules for Operant Learning

Thus far we have shown that in vitro data-derived dopamine-modulated STDP functions are sufficient to generate putative D1 and D2 MSN responses over the course of an operant-learning task. We now ask to what extent this complex set of non-standard STDP functions (Figure 4) are necessary to generate such responses: that is, could the complexity of the three-factor dependency (on receptor type, dopamine concentration, and spike-timing) be explained by the need to generate a particular set of MSN responses?

To address this, we performed an exhaustive, “brute-force” search in the 4D parameter space of plasticity coefficients for each MSN type. Full details are supplied in the Methods but, briefly, each search was divided into two stages: a first stage with an extensive parameter range, followed by a more focused search around the best-fitting responses. For each set of plasticity coefficients encountered, we ran a set of the simulated learning experiments to obtain spike count profiles. We then used a feature-based method to define a score to determine how well the profiles matched the targets in Figure 1.

**Figure 9:**
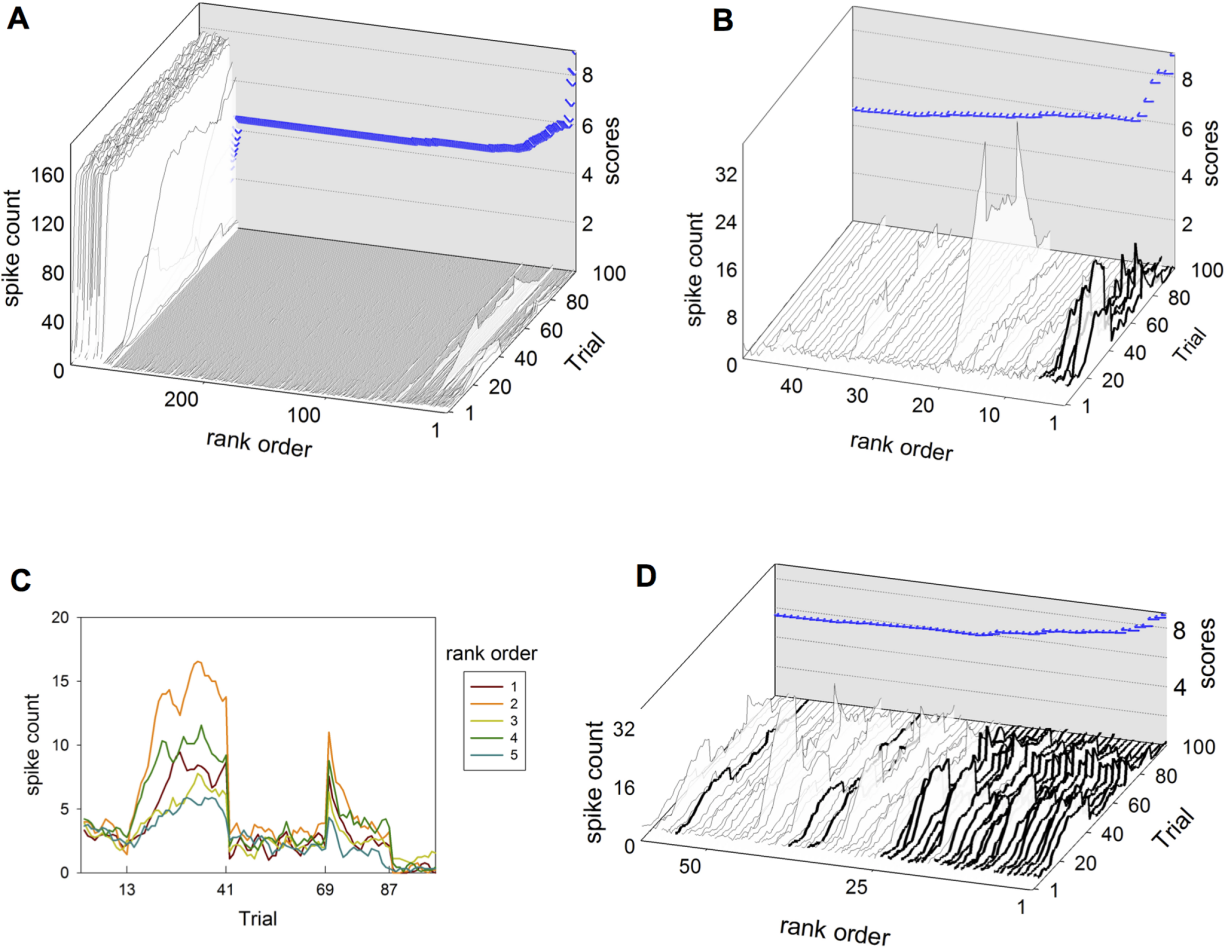
Exhaustive search of plasticity coefficients for the STDE model, seeking D1-MSN spike count profiles for operant learning (see **Figure 1**). (A) The best scoring 300 (out of 625) D1-MSN activity profiles from a broad search across the entire parameter range. (B) The top 50 ranked profiles from (A); note the much smaller range of spike counts on the vertical axis compared with (A). (C) The top five profiles from this coarse-grained search (heavy lines in (B)) used to define a more focused, fine-grained search. (D) The top-scoring 60 D1 MSNs from the focused search. The best 26 were deemed good matches by visual inspection (shown in heavy lines) and their coefficients constituted the discovered set.

**Figure 10:**
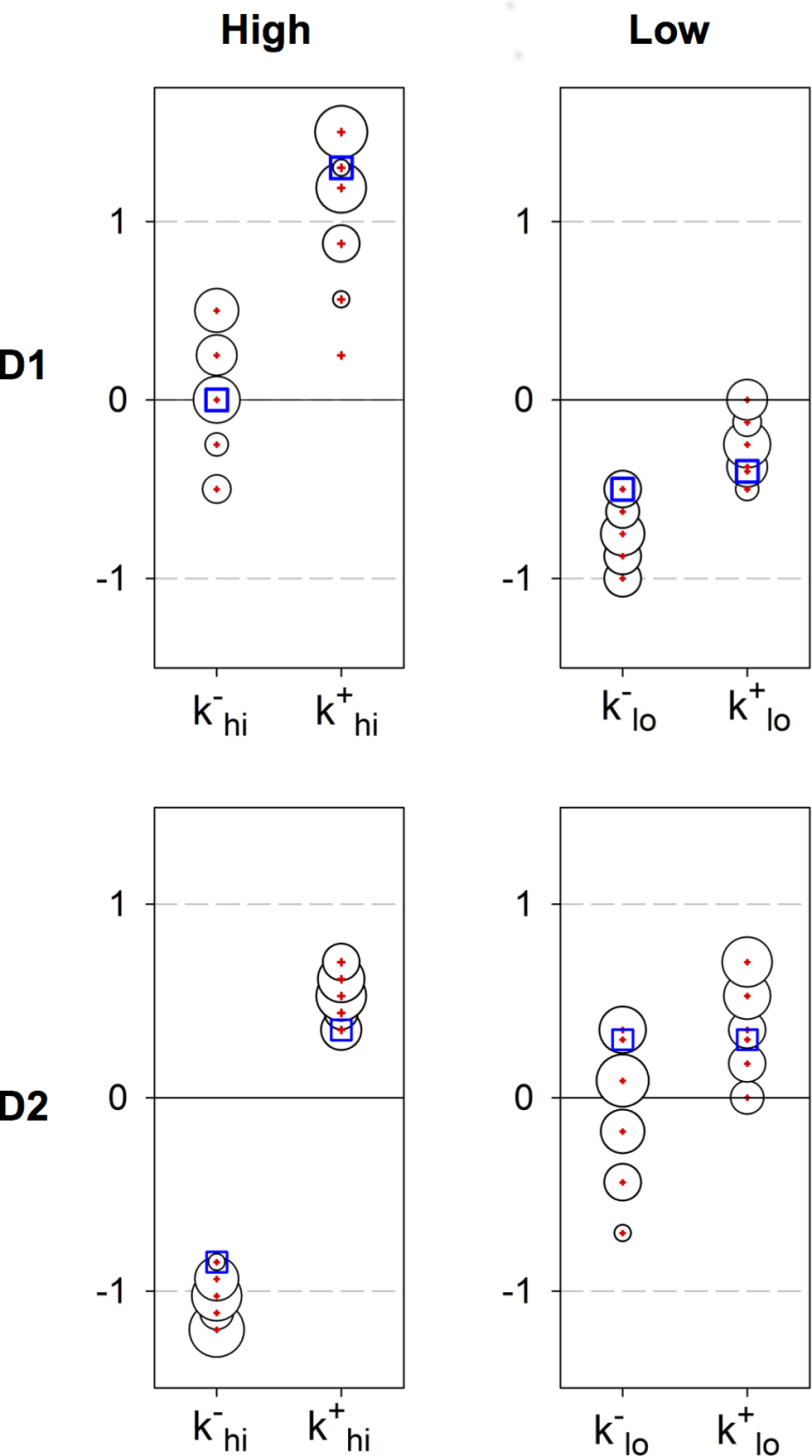
Results of exhaustive search of plasticity coefficients for the STDE model. The plot layout corresponds to that in Figure 4. In each plot, the red crosses show the coefficient value, the area of the bubble is proportional to the number of times that value was found, and the blue squares are the hand-chosen values used to create the activity profiles in Figure 7. The discovered set for D1-MSNs comprised the 26 best profiles from Figure 9D and are reported in the top row. For D2 MSNs, there were 32 candidates with satisfactory profiles at the end of both coarse and focused searches; they yield the plots in the bottom row.

**Figure 11:**
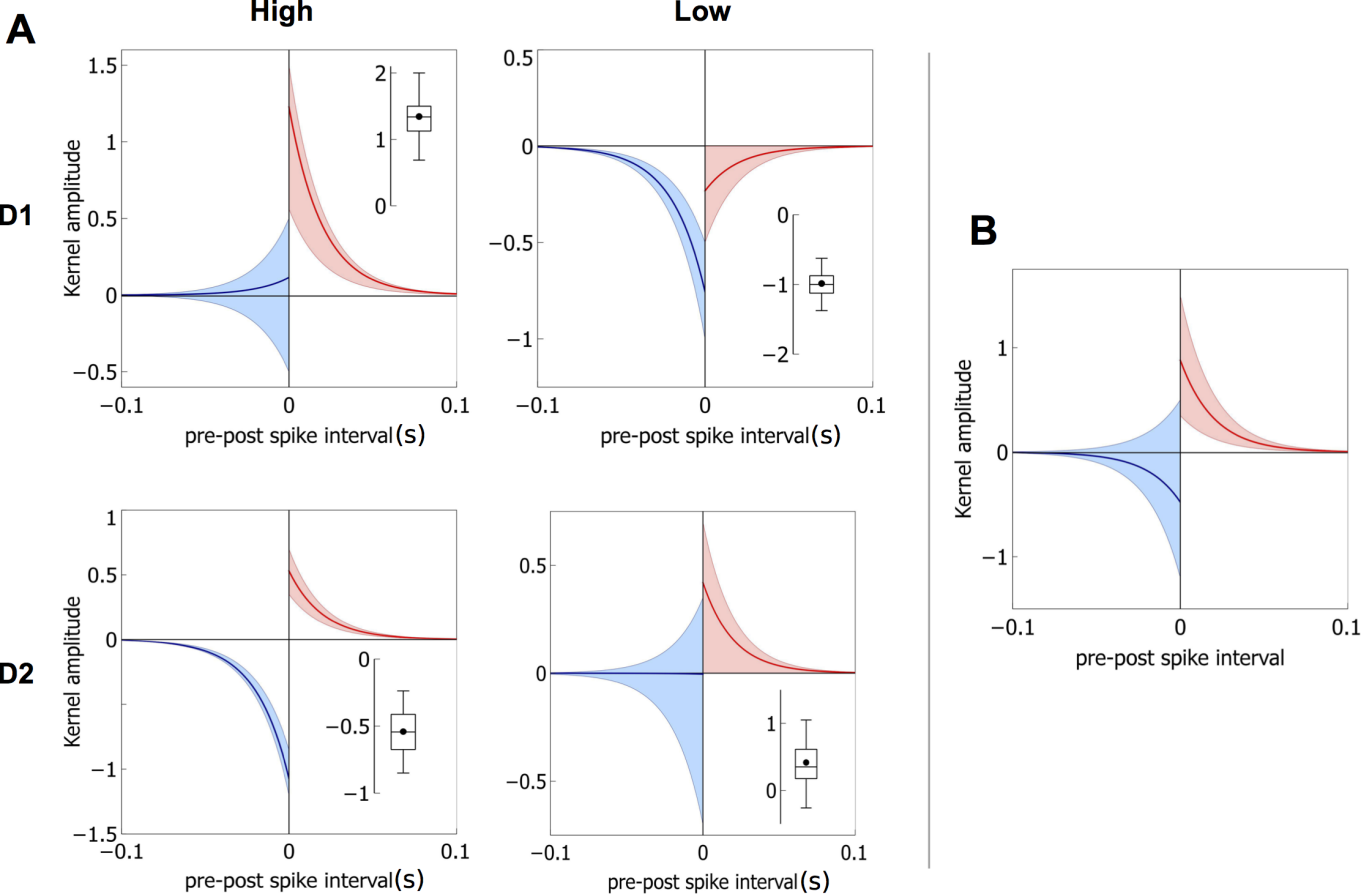
The plasticity rules obtained under the exhaustive search with the plasticity coefficients of **Figure 10**. In (A), each plot shows the range of the resulting plasticity functions for positive and negative spike-pair timing in pale red and blue, respectively, with the mean shown by solid lines. The plot layout is the same as that used in Figures 4 and 10. The box-and-whisker inset plots show the quartiles, medians, extrema, and means (black dots) of the sum 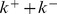, for each function pair in Figure 10; this gives a measure of the overall direction of plasticity, given a random sampling of pre-post spike pair timings. (B) The extent and mean of the plasticity functions at high dopamine levels across D1 and D2 type MSNs combined (from left column of plots in (A)).

Figure 9 illustrates the search process, and the diversity of activity profiles encountered for D1 MSNs. Figure 10 shows the range of satisfactory plasticity coefficients discovered by the search for both MSN types. Figure 11 shows the range of STDP functions resulting from the distribution of values for each plasticity coefficient that gave good matches to the MSN response profiles. Across the three factors of spike-timing (negative, positive), MSN type (D1, D2), and dopamine level (low, high), six of the eight functions were always restricted to the same sign (LTP or LTD) as the data of Shen and colleagues [17]. Thus, our model predicts that the dependencies on timing, dopamine-level, and dopamine-receptor for these STDP functions are necessary for the putative MSN response profiles under operant conditioning.

However, we also predict some diversity in the necessary learning rules for two functions with negative spike-timing (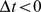). For D1 MSNs at high dopamine levels (Figure 11A, top left panel) our model predicts the possibility of either LTP or LTD for 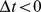. The overall sign of plasticity, averaged over randomly chosen pre-post spike timings, is determined by the sum 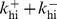, shown in the plot inset. For D1 MSNs at high dopamine, we therefore predict an overall LTP-like outcome. For D2 MSNs at low dopamine levels (Figure 11A, lower right), our model also predicts the possibility of either LTP or LTD for 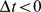. However, once again, the overall direction of plasticity is almost always (with one outlier) LTP-like with 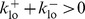.

### STDE Plasticity Model Replicates Experimental Results on Cortico-Striatal Plasticity

We derived our cortico-striatal plasticity model by extrapolating and combining Pawlak and Kerr’s [22] report of STDP at cortico-striatal synapses and Shen and colleagues′ [17] data on that plasticity’s dependence on dopamine receptor type, concentration, and the sign of spike-timing, and extending to include arbitrary levels of dopamine and an eligibility trace. Here we answer the question of whether this extrapolated and extended model can capture these underlying data.

In Figure 11B we plot the range of STDP kernels predicted by the sets of successful plasticity coefficients from our exhaustive search if, as in the study of Pawlak and Kerr [22], D1 and D2 MSNs were indistinguishable. We find that the mean kernels give the classic STDP profile and some evidence of LTP at negative spike timings, exactly replicating Pawlak and Kerr’s [22] result.

To check that our models could replicate the results of Shen and colleagues [17]—shown in the insets in Figure 4—we simulated their plasticity induction protocols at a single AMPA synapse of the spiking MSN model using the full STDE model. Each condition of D1 or D2-type MSN, “high” or “low” dopamine, and positive or negative spike-pair timing was simulated; details are given in the Methods. The outcomes of the experiment were a set of EPSP-ratios, one per condition, comparing the EPSPs before and after the period of plasticity induction.

**Figure 12:**
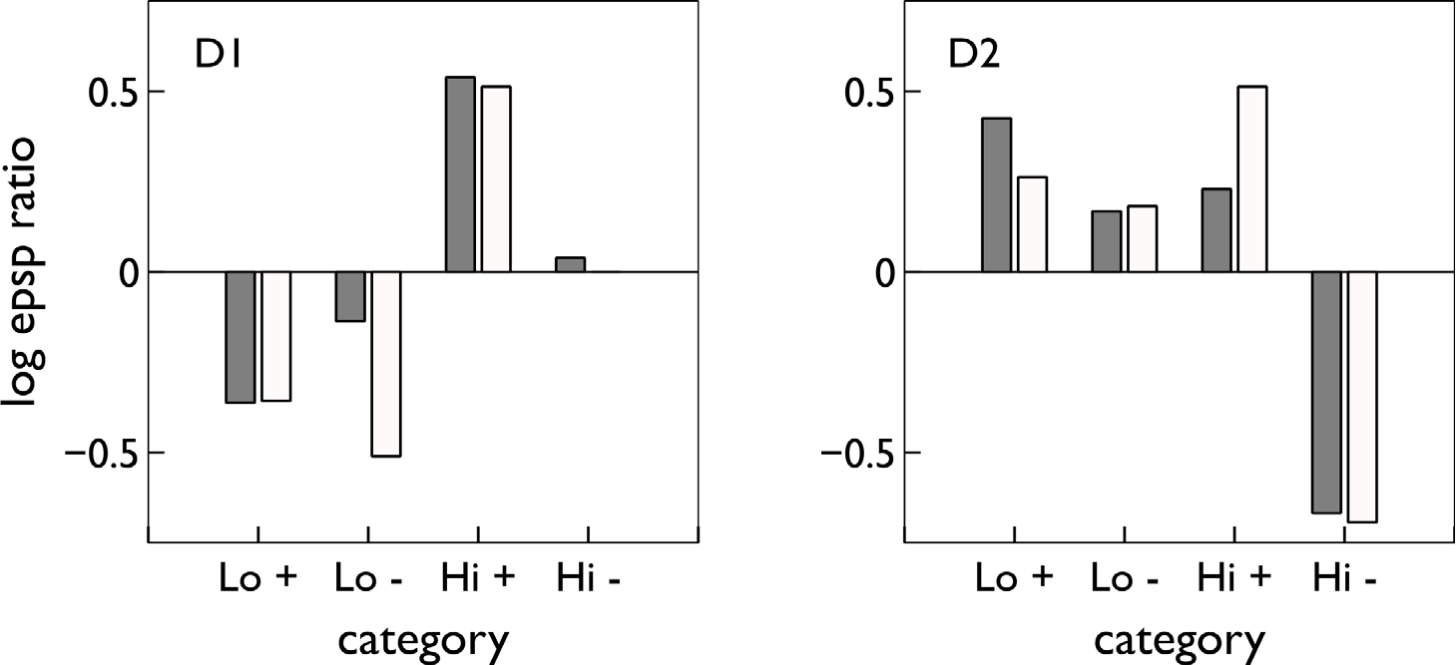
STDE model’s replication of Shen and colleagues’ plasticity results (plotted in the insets of **Figure 4**). Each bar is the log of the ratio of final-to-initial EPSP amplitude after a period of plasticity induction. Solid/open bars are for the model/data respectively and each condition refers to a level of dopamine concentration (“hi” or “lo”) and spike pair timing (+/− for positive/negative pre-post timing). For each of D1- and D2-MSNs, plasticity coefficients were drawn from the sets deemed successful in the exhaustive search. In each case (D1 or D2) the four outcomes were uniformly scaled (under linearity of learning) to the best least-squares fit.

We simulated such a complete experiment using different sets of successful plasticity coefficients found by the exhaustive search. Figure 12 plots the EPSP-ratios for the data against those obtained using a typical set of coefficients, showing that the sign of plasticity is preserved in all cases and several of the rank-order relations between pairs of experimental conditions are preserved. Thus, the plasticity model parameters necessary for successful action selection and suppression in an operant task are consistent with in vitro data on plasticity at a single cortico-striatal synapse.

## Discussion

A central hypothesis in reinforcement learning is that cortico-striatal synapses are the neural substrate for the interface between action and reinforcement. While a commonly held idea, a comprehensive quantitative model is necessary to test this hypothesis by showing that the properties of cortico-striatal plasticity can successfully bridge the levels from plasticity at individual synapses, to the changed activity of the whole neuron, the effects on its neuronal network, and the consequences for behaviour. Bridging these levels requires solving the challenges of understanding: (i) the roles of the complex dependence of cortico-striatal plasticity on dopamine level, postsynaptic receptor type and spike-timing; (ii) how to integrate the different time scales of plasticity (10–100 ms) and environmental feedback (1 s); and (iii) how both the plasticity rules and time scales fit with the functional dichotomy of the D1 and D2 MSN pathways in action selection.

We proposed here a comprehensive modelling framework for testing this hypothesis, which links dopamine-modulated cortico-striatal plasticity [6],[8],[15],[17], phasic dopamine signals carrying environmental feedback [2],[4],[37],[38], and the striatum’s role in reinforcement conditioned action selection [12]–[14].

To tackle the first challenge, we have proposed a cortico-striatal plasticity model that can accommodate the most extensive in vitro data currently available for this phenomenon [17],[22]. From the data we inferred that the rules governing cortico-striatal STDP vary independently along two dimensions: neuron type (D1/D2 MSNs) and the level of dopamine. The resulting plasticity rules show continuously varying STDP functions obtained by mixing those at the extremes of high and low dopamine levels. To tackle the second challenge, we extended this model to incorporate STDE, using an eligibility trace to bridge the delay between the action-representing signals from cortex and the subsequent change in dopamine level caused by the action’s outcome. To tackle the third challenge, we used a full model of the basal ganglia network to find the relative balance of D1 and D2 MSN responsiveness required for optimal selection and for optimal suppression of an action. From this we predicted how the activity of D1 and D2 MSNs should change over an operant learning task in order to optimise selection after learning and suppression after extinction of a single action. While these predictions are consistent with the broad hypothesis that the direct pathway from D1 MSNs promote selections and the indirect pathway from D2 MSNs suppresses selection [18]–[20], our network model revealed the new hypothesis that D1 and D2 MSNs coding the same action co-operate to produce optimal selection. This hypothesis is, of course, predicated on there being single populations of both D1 and D2 MSNs representing single actions. Though this is a widely held assumption [13],[20],[25],[51], it is an open question as to whether it is the case, or whether D1 MSN populations represent specific actions and D2 MSNs are recruited more generally to contribute to the inhibition of a set of actions [52].

We found that the profiles of D1 and D2 MSN activity changes can be generated in full by the in vitro derived plasticity rules using only a delayed phasic dopamine signal as feedback, a remarkable convergence of a high level theory of learning and in vitro data that, at first glance, have no clear functional implication. Unexpectedly, the data-derived plasticity rules caused a small increase in the action-coding D2 MSN activity over learning, supporting the new hypothesis of D1-D2 co-operation for optimal selection. Also remarkable was that most of the complex dependencies of cortico-striatal plasticity on spike-timing, dopamine level, and MSN-type were not only sufficient but necessary to generate the D1 and D2 MSN activity profiles over learning and extinction (Figures 10 and 11).

### Limitations on Interpreting In Vitro Plasticity Data

In going from in vitro data to learning rules, some interpretation of that data was clearly necessary. For example, we adopted the naturally occurring level of dopamine in the in vitro experiments as the nominally “high” value in setting function parameters. The precise levels of dopamine here may not correspond with the highest values accessible in vivo but this is not critical. Rather, we assume that the trend in parameters is monotonic with dopamine level so that the data determine these trends rather than the values per se. The monotonicity assumption is a key aspect of our framework and more experimental work is required to establish if this is the case.

While the data of Shen and colleagues [17] form the most complete picture of the factors controlling cortico-striatal plasticity, our extrapolation to the set of STDP kernels (Figure 4) is based on a particular interpretation of their experimental protocol. They used an asymmetric stimulation protocol with three postsynaptic spikes preceding each pre-synaptic spike in the negative timing condition, but three pairs of pre- then postsynaptic spikes in the positive timing condition, each pair spaced by 15 ms. Thus their positive-timing protocol contains both positive and negative delays, implying that it contains contributions from both positive and negative STDP kernels. In our interpretation, we simplified this by assuming the positive-timing protocol was predominantly receiving contributions from the positive STDP kernel (Figure 4). Nonetheless, it was encouraging that our unconstrained search returned kernel coefficients with the signs we extrapolated from the Shen and colleagues’ data, and recovered the generic MSN STDP kernel reported by [22].

A further common limitation for any extrapolation from in vitro work to in vivo application is that many of the in vivo-like conditions are intentionally removed during in vitro studies to provide close control over the experimental question at hand. For the Shen and colleagues’ [17] data, these include the injection of current to hold the membrane potential close to −70 mV, thus minimising the impact of NMDA receptors, and the use of GABAa antagonists to prevent any effect of inhibition (which may play a key role in STDP [53]). Despite these limitations, we showed that the single spiking MSN models with our plasticity rules could produce the required activity profiles over an operant task even though they incorporated input to both NMDA and GABAa synapses.

Also missing in vitro are the dynamics of the intra-striatal signals in vivo that may directly or indirectly affect plasticity at cortical synapses on MSNs, particularly those originating from the interneurons. As well as GABAergic signals from the fast-spiking interneurons, cholinergic interneurons may play a dual role through both postsynaptic modulation of plasticity [54] and the shaping of dopamine release in the striatum [55]. Thus, a complete systems model of cortico-striatal plasticity will require the integration of synaptic and network level contributions.

Finally, STDP is a phenomenological description at the level of spikes of a set of intra-cellular signalling processes, and more detailed modelling of those processes (e.g., [56]–[59]) will be essential to shed light on the effects of spiking history, of dopamine’s triggering of intra-cellular signalling cascades, and particularly on the discontinuity at 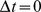.

### Interpreting the Phasic Dopamine Signal in Ethological Action Learning

The plasticity rules developed here are consistent with a range of interpretations of the origin of the phasic dopamine signal. They are consistent with the dominant hypothesis that phasic firing of dopamine neurons encodes a reward prediction error [2],[3],[5],[37],[38]. However, we note that they are also consistent with our recent proposal that phasic dopamine is, in part, associated with a sensory prediction error that can enable intrinsically motivated action discovery [4],[60]. Here, serendipitous interaction with the environment to effect some predictable outcome therein, can cause learning of the contingency between action and outcome. Recently [61] we have tested the ability of the plasticity rules developed here to effect action discovery by embedding a model of the basal ganglia, equipped with these rules, in a simulated behaving agent that can learn simple action outcome associations. The agent was able to successfully learn the associations and, moreover, the specific plasticity rules described here demonstrated superior performance to a range of plausible alternatives.

### Relation to Models of Learning in Basal Ganglia

There have been numerous attempts to model the learning taking place in basal ganglia and that identify the locus of plasticity as the cortico-striatal connections. Many of these models use a temporal difference (TD) learning rule or variants therein; for a recent review see [62]. The learning signal in TD algorithms is an “error” or discrepancy between a predicted reward and the actual value received. The error is derived from algorithms grounded in machine learning [63], but, in biological terms, it is often identified with phasic dopamine [2],[64]. In contrast, we have no algorithmic origin for phasic dopamine because our account does not address this level of description (the dynamics of dopamine are described phenomenologically).

Nevertheless, we might, in principle, attempt to map components of the TD “rule” onto mechanisms we have described here. This exercise would probably fail however, as the the TD rule is not inherently of the three-factor kind in which our framework sits; that is, it does not explicitly include pre- and postsynaptic firing, and an error/dopamine modulatory term. The difficulties encountered with mapping TD in this way have been discussed at length by Worgotter and Porr [65]. However, this does not preclude our plasticity framework from supporting operant learning in which phasic dopamine is obtained algorithmically from internal models of prediction. Indeed, we have recently demonstrated such a model in complete cortico-basal ganglia-thalamic loops, embodied in a behaving agent [61]. This model showed how our plasticity rules have rate-coded (non-spiking) equivalents that are part of the well-known BCM family of learning rules [66],[67]. This was made possible because of the intimate relation between BCM rules and STDP [68].

### Cortico-Striatal Plasticity in Goal-Directed and Habit Learning

A key distinction in instrumental learning tasks is made between goal-directed and habitual behaviour. An animal expressing goal-directed behaviour modifies that behaviour in response to a change in the value of its outcome or in the contingency between the action and the outcome; one expressing habit behaviour does not [9],[69],[70]. The inference is then drawn that goal-directed animals have access to explicit representations of outcomes linked to actions to guide behavioural choice, which are updated after changes to the outcome irrespective of performing the action. By contrast, habitual animals make behavioural choices on the basis of stimulus-response pairings and can only update this association after repeatedly performing the action cued by the stimulus [69],[71].

Habitual and goal-directed behaviour have been respectively linked to the dorsolateral and dorsomedial striatum [9]–[11],[72]. Lesioning the dorsolateral striatum [73]–[75] or disrupting dopamine signalling within it [76] prevent habit formation. Correspondingly, there is a re-organisation of single neuron activity in the dorsolateral striatum during habit formation [26],[27],[29],[75]. Lesioning the dorsomedial striatum [74],[75],[77] prevents sensitivity to devaluation or contingency changes. Recent studies of comparative plasticity have shown that only the dorsomedial striatum has evidence of synaptic plasticity unique to goal-directed learning [78],[79]. Together, these data raise the key question of what differs between circuits containing the dorsomedial striatum and dorsolateral striatum that ultimately results in goal-directed and habitual behaviour [71].

Our model framework here has three separate components: (1) models of the signals from cortex and of dopamine release, both per trial and their changes over trials; (2) a synaptic-level plasticity model (dopamine-dependent STDP); and (3) a circuit-level action selection model. Any or all of these could be a source of difference between dorsomedial and dorsolateral striatum, and hence candidates for the difference between goal-directed and habitual behaviour. We consider the first two here, as basal ganglia circuitry is well-conserved between regions [80] (but see [81]) and it is not immediately clear how differences in the action selection mechanism could differentiate between outcome-driven and stimulus-driven behaviour.

Together, model components 1 and 2 reinforce an action by increasing the probability of its selection on a subsequent trial, and do this by increasing the influence of a fixed salience signal from cortex over the basal ganglia selection process. In this respect, the model mechanisms are neutral as to whether the action request from cortex is primed by a representation of the outcome to follow (goal-directed) or a representation of the preceding stimulus (habitual). However, for simplicity we assumed throughout that the input from cortex had the same salience on every trial whether the outcome was delivered or not, and so did not reflect changes in value. Thus, our model of inputs is currently consistent only with stimulus-response behaviour, and therefore our model framework as a whole is most consistent with the dorsolateral striatum. Nevertheless, within this framework, component 2 (the synaptic-level plasticity model) remains neutral to the goal/habit distinction.

Extending our model framework to account for goal-directed behaviour would require identifying where information about value or contingency become encoded. Dorsolateral and dorsomedial striatum receive inputs from different cortical regions [82] and so one possibility is that only the action-request inputs to dorsomedial striatum encode value and contingency information. One candidate here is orbitofrontal cortex: it projects to the dorsomedial striatum [83], its neurons’ activity represents the expected value of an action [84],[85], and optogenetic stimulation of its projection neurons promotes the maintenance of action during extinction [75] consistent with their encoding of value. In this view, changes to value or contingency update their representations in cortex and are reflected in the changed salience of the action request to striatum, allowing for more rapid changes to behaviour than could occur solely via synaptic plasticity.

A particular challenge for this view are non-contingent reinstatement phenomena where an action is immediately re-energised after extinction by a single non-contingent presentation of its pre-extinction outcome [86]. For if goal-directed behaviour is driven by the rapidly diminishing salience of an action during extinction, then reinstatement forces us to assume that a single outcome presentation is sufficient to restore that salience.

Another possibility is that the dopamine signal is not the same in dorsomedial and dorsolateral striatum, as we have assumed here. Separate midbrain dopamine systems project to these regions [81],[87],[88]. Reflecting this, intact dopamine signalling in dorsolateral striatum is necessary for the formation of habitual behaviour [76], and blunting dopamine signalling prevents the formation of habitual behaviour but does not prevent goal-directed behaviour [89]. In this view, changes to value and contingency would be reflected by the evoked dopamine signal in dorsomedial striatum and not in dorsolateral striatum, and thus appropriately modulate cortico-striatal plasticity only in dorsomedial striatum. Particular challenges for this view are that dopamine signals to the striatum seem to encode the same information everywhere [90] (but see [91]) and the speed of change—if behavioural change depends solely on synaptic plasticity, then behaviour is likely altered slowly but the goal-directed system seems to rapidly adapt [71].

A further possibility (which challenges our synaptic-level neutrality) is that dopamine-dependent STDP is different between the dorsolateral and dorsomedial striatum, so that even with the same input signals (cortical and dopaminergic), the cortico-striatal weights are updated differently between the two regions. There is good evidence that synaptic weight change differs between the two regions in both skill-learning [92] and goal-directed learning [79], though these data cannot distinguish between whether the inputs differed, thus differentially recruiting the same plasticity mechanism, or the mechanism of plasticity itself differed. Consistent with the latter, in vitro work has suggested differences in high-frequency stimulation induced LTP between medial and lateral striatum [93]. In this view, for the synaptic plasticity rules themselves to reflect changes to outcome in dorsomedial and not dorsolateral striatum, it follows that the outcome-related signals (cortical and/or dopaminergic) must be input to both areas, but that the plasticity mechanisms are sensitive to changes in these inputs only in dorsomedial and not dorsolateral striatum. Again a particular challenge for this view is the speed of behavioural change for goal-directed behaviours if they are solely dependent on synaptic plasticity and not on computations performed elsewhere [71].

The above ideas are naturally speculative, reflecting the current lack of data on the precise relationship between different forms of behaviour and the details of cortico-striatal plasticity in different striatal regions [70]. A contribution of our model framework is that by bridging the levels from a single synapse to overt behaviour it provides a basis for framing the alternative hypothesises and their implications.

### Implications for Cortico-Striatal Plasticity

Our search for the necessary plasticity coefficients to generate the D1 and D2 MSN activity profiles predicts that two of the eight coefficients could be positive or negative (Figure 11). Thus, for D1-MSNs at high levels of dopamine and for D2-MSNs at low dopamine levels, there is a possibility of LTD or LTP for negative spike-pair timing. This apparent ambiguity may be resolved in two ways: (i) that there is a corresponding variation of plasticity rules across individual MSNs (or even individual synapses) in an individual animal brain; or (ii) that these rules are subject to constraints that lie outside our framework, and thus in vivo all combinations of LTP and LTD are those we inferred from the Shen and colleagues′ [17] data (Figure 4). Such constraints could include that the specific dopamine-activated intracellular signaling pathways that ultimately give rise to changes in plasticity can allow only a single direction of change for a given combination of dopamine receptor and level, and consequently can only express one of LTD or LTP at a single synapse for that combination.

We hypothesised that extinction in operant learning involves active suppression of the action by D2 MSNs, not (solely) unlearning of the action at cortico-striatal synapses onto D1 MSNs. While this is compatible with modern theories of behaviour that posit that extinction is not a simple unlearning of previous competence [46], it leaves open the question of how post-extinction phenomena of spontaneous recovery of action can occur if the action is actively suppressed. We showed our model nonetheless could account for both phenomena of contextual renewal (immediate recovery of extinguished action in new context) and reacquisition (rapid re-learning of extinguished action). This occurred because, in extinction, we predict that D1-MSN synaptic conductances would regress to their original untrained state only when extinction and post-extinction testing were in the same context, and so a change of context allows rapid recovery of action. Thus in our model spontaneous post-extinction recovery arises solely from the plasticity rules without recourse to additional hypotheses such as state-space splitting proposed by the model of Redish and colleagues [94].

The complexities of cortico-striatal plasticity’s dependence on dopamine receptor-type, dopamine level and spike-timing mean that inferring the effect of changes in these factors is fraught with difficulty, and models are necessary to guide us. Simplifying such models in turn provides us with useful heuristic guides. On the basis of the data available at the time, Reynolds and Wickens [15] sketched a widely used and valuable heuristic guide to the overall direction of weight change at cortico-striatal synapses as a function of dopamine concentration (see Figure 4 in [15]). Our data-derived cortico-striatal plasticity model predicts a smooth morphing of STDP kernels with changing levels of dopamine, switching gradually from LTP to LTD. We can thus use our model to update the heuristic guide to the dopamine-dependence of plastic change, and importantly separate the effects on D1 and D2 MSNs.

**Figure 13:**
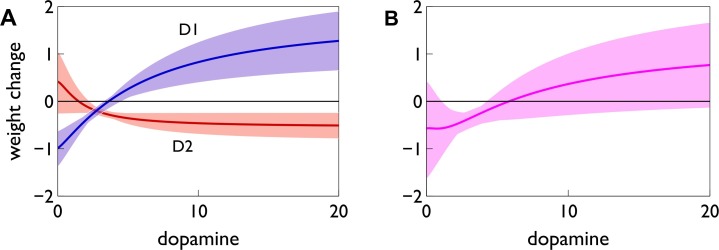
Expected overall weight change as a function of dopamine concentration. Here we plot the mean (line) and range (shading) of the overall weight change (sum of the plasticity factors 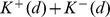) at a given dopamine level 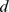, across every set of plasticity coefficients found by the search (Figure 10). (A) Separate plots for the found sets of D1 and D2 MSN coefficients, showing the dopamine dependence of each neuron type. (B) The sum of the individual MSN-type contributions in (A).

In Figure 13 we plot the sum of the STDP kernel amplitudes as a function of dopamine concentration, which approximates the expected overall weight change for random trains of input and output spikes, for every successful coefficient set from the exhaustive search. The range of weight changes shown are hence consistent with successful action selection and suppression of the key action. We see that, if we plot the equivalent curve to that in [15] by not distinguishing D1 and D2 MSNs, then our model predicts that the average total measured weight change approximates the curve in [15]. However, the range of total weight change we observed, consistent with successful selection of the key action, covers both LTD and LTP at many dopamine levels. This is accounted for in the model by its prediction that increasing dopamine switches D1 MSN synapses from LTD to LTP and D2 MSN synapses from LTP to LTD. Our results thus suggest that the dependence on both dopamine receptor and dopamine concentration forms the minimal model of cortico-striatal plasticity.

## Methods

### Rate-Coding Model of the Basal Ganglia Network

Figure 2A shows the basal ganglia network implemented by the model (see [34],[80],[95] for a detailed discussion of assumptions behind this architecture). Each action is encoded in a discrete “channel” throughout the model. Within each nucleus, each channel is represented by a single, rate-coded leaky-integrator unit whose output stands for the mean activity of a population of neurons that might instantiate the channel in vivo.

The assumption of a channel architecture is based on the long-standing concept of parallel anatomical loops running throughout the basal ganglia nuclei [96],[97]. Both anatomical and electrophysiological evidence points to the existence of channels representing discrete actions. For example, the somatotopic map found within the striatal motor territory is maintained throughout the basal ganglia circuit, such that there are separate channels for arm, leg, and face representations [18],[98]. Similar topographic maps have been proposed for the other macroscopic channels [18]. Moreover, within these limb representations, there are discrete channels corresponding to particular movements, demonstrated in striatum by microstimulation [99] and markers for metabolic activity during behavior [100]. Recently, Fan and colleagues [101] provided a compelling demonstration that basal ganglia output neurons coding for selection of the same action are physically clustered, just as predicted by the channel architecture.

Cortical input to each channel represents the “salience” of that action. In general, the salience of an action at any given moment will depend on the integration of diverse information on current motor commands, sensory information, and context by convergent inputs to individual MSNs [13],[80],[102]. For the rate-coding model of the basal ganglia network, we collapse this into a single scalar value for the salience of the represented action, as we are interested in the ability of the network model to perform selection or suppression on the basis of this salience signal, not in how that signal is computed. Consistent with this assumption, a recent optogenetic study has shown that selecting an action is controlled by the activity of cortico-striatal neurons in sensory cortex [103]. For the spiking MSN model, we explicitly represent changes in context by altering the sub-set of active cortical inputs (detailed below), and thus simulate how salience is dependent on context.

Competition between channels for behavioural expression is provided in a “selection pathway” comprising D1-MSNs, STN, and the output nuclei that form a feedforward, off-centre, on-surround network. The circuit with STN, D2-MSNs, and GPe acts to moderate the overall levels of excitation and inhibition in the selection pathway and also perform action suppression for individual channels (Figure 2B).

The average activity 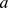 of all neurons comprising a channel’s population changes according to 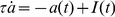
(1)


where 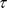 is a time constant and 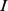 is summed, weighted input. We used 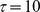 ms throughout. The normalised firing rate 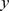 of the unit is given by a piecewise linear output function 
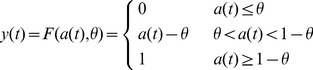
(2)


with threshold 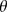. Negative thresholds thus ensure spontaneous output, which we use to ensure STN, GPe, and GPi/SNr have tonic output (see below).

The following describes net input 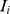 and output 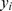 for the 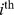 channel of each structure, with 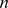 channels in total. The full model is given by [35]:

Striatum D1: 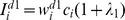,
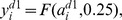



Striatum D2: 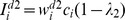,
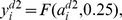



Subthalamic nucleus: 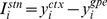,
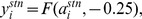



Globus pallidus external segment: 
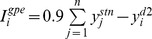


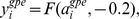



GPi/SNr: 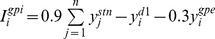,
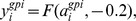



Each cortical signal 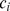 simulating an action request was input to channel 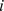 in the D1-MSN, D2-MSN, and STN populations. The network model included opposite effects of activating D1 and D2 receptors on MSN activity: D1 activation facilitated cortical efficacy at the input, while D2 activation attenuated this efficacy [45],[104],[105]. Thus, if the relative activation of D1 and D2 receptors by tonic dopamine are 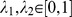, then the increase in efficacy due to D1 receptor activation was given by 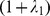; the decrease in efficacy due to D2 receptor activation was given by 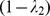.

In the implementation used here, the model had six channels but only two were actively driven by cortical input. The other channels are required, however, as they have quiescent firing rates in STN and GPe that contribute to overall activity.

### Establishing Relative D1 and D2 MSN Responsiveness for Selection and Suppression

We used this model to predict the relative responsiveness of D1 and D2 MSNs that optimised selection of an action (emulating the target situation at the end of the learning epoch) and, separately, that optimised the suppression of an action (emulating the target situation at the end of the extinction epoch). The ability to select a particular action can only by tested with reference to at least one other possible alternative action, so we considered two competing signals, one signal representing a fixed “control” action, available for selection throughout, and another signal representing the key action learnt and extinguished over the course of the experiment.

We input this pair of salient signals (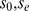) to two channels in the model, respectively termed the control (subscript 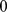) and experimental channel (subscript 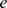). For a given pair of inputs, we read out the outcome of the competition from the output of the basal ganglia 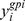 (SNr/GPi in Figure 2): 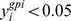 signalled a sufficient fall in GPi’s tonic inhibition for selection of the corresponding action on channel 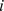.

Each input pair thus had four possible outcomes: no selection, control channel selected, experimental channel selected, or dual selection. The ideal selector outcomes were then defined as follows. For both learning and extinction we demanded that no action be selected if both inputs (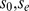) were less than the MSN output threshold 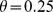. After action learning we required that, if 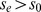, then the experimental channel is selected, and if 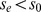, the control channel is selected; if 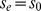, then no selection is required. After extinction of a previously learned action represented by the experimental channel, we required that that channel is never selected no matter what the value of 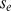
—representing suppression of that action—and that the control channel is selected if 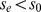.

The salience pairs 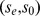 were constructed by allowing each of 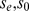 to range over a set of ten discrete values in the interval 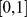. The set of ideal outcomes (for each of learning and extinction) over all 100 salience pairings constitutes an ideal selector template for model comparison, and these are plotted in Figure 3 for learning (Figure 3A) and extinction (Figure 3B), with experimental and control channels being identified with channels 1 and 2, respectively. For each of the 100 input pairs, the input on the experimental and control channels occurred at *t* = 1 s, and *t* = 2 s, respectively. The GPi output was read out at equilibrium, and the simulation time-step was 0.01 s. Over all 100 input pairs, the model performance was then compared to the template, and summarised as a percentage match.

The ability of the network model to match these two templates was tested by varying the relative “responsiveness” to input of the D1 and D2 MSN populations of the experimental channel. Responsivess is defined here as the ratio of the input to output value for the population. As both the cortico-striatal input weights and the level of tonic dopamine affect responsiveness, for this channel alone we set 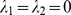 and varied the D1 (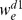) and D2 (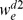) MSN input weights independently over the range 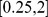. To allow us to investigate a full range of MSN behaviour, we dropped the saturation requirement on the output (condition (iii) in Equation 2). For the control channel, we set 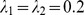 and the input weights to 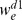  =  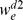  =  1, following our prior models [35].

### Formal Description of the Plasticity Framework

Here, we give details of the plasticity framework that incorporates the three factors of postsynaptic neuron type, dopamine concentration, and spike-timing at the scale of STDP. All parameters are collected together in Table 1.

**Table 1:** Parameters for mixing STDP functions (kernels) and plasticity rules.

Parameter Group	Parameter	Symbol	Value(s)
D1 Mixing function 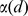	Naka-Rushton exponent	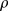	1.2
	Naka-Rushton threshold	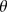	6.0
	maximum value	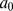	1.2
D2 Mixing function 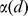	Naka-Rushton exponent	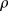	1.4
	Naka-Rushton threshold	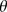	1.8
	maximum value	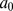	1.0
Timing and learning rates (common to both D1- D2-MSNs)	kernel 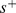 time constant	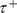	0.02
	kernel 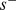 time constant	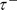	0.02
	eligibility time constant:	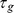	0.3
	learn rate	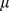	0.65

### From In Vitro Data to STDP Functions

We start by assuming constant dopamine and STDP (no eligibility). Let 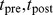 be a pair of postsynaptic and presynaptic spike times respectively, and put 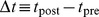. For each of the two classes, D1-, D2-MSNs we define STDP functions (kernels) for the following four cases: 
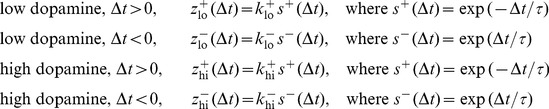
(3)


### Extending the Model to Arbitrary Levels of Dopamine

We define functions 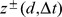 for any 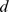, by “mixing” the functions at the extremes of the range, 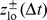 and 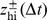 (see Figure 5). We use a simple linear blending scheme 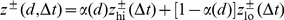
(4)


where the mixing functions 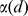 for each of D1- and D2-MSNS are shown in Figure 5D. It is conveniently expressed by a Naka-Rushton equation 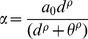
(5)


but no special significance is assigned to this form; all that is required is a rapidly increasing, then saturating, monotonic function of 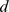 with no point of inflexion.

The parameters 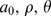 were chosen to ensure: (i) 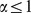 over the range of dopamine level used; (ii) that, for each of D1- and D2-MSNs, with typical plasticity coefficients consistent with the data in [17], there is little or no overall plastic change at tonic levels of dopamine.

In extending the formalism further to incorporate eligibility (next section), it is useful to rewrite (4) in an alternative form 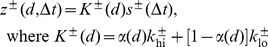
(6)


We refer to the 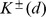 as “plasticity factors,” and plot them in Figure 5C and 5E.

For STDP, the resulting change in synaptic weight 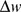 due to a single pre-post spike pair is given by 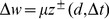
(7)


where 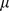 is a learning rate.

### Extension to Longer Time Scales: Spike Timing Dependent Eligibility

We base our eligibility trace model on that of Izhikevich [44], extending to incorporate arbitrary levels of dopamine, and testing its application across all forms of non-standard STDP we observe for cortico-striatal synapses. The basic idea is that each spike pair creates a step-change contribution 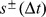 to a corresponding eligibility trace 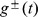, where 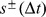 are the normalised STDP functions defined in (3), and the positive/negative sign applies according to whether 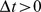 or 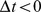. The step change for either can be positive or negative, corresponding to a potential increase (LTP) or decrease (LTD) in synaptic weight. The eligibility decays exponentially with time constant 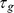, so the eligibility 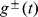, due to a single spike pair, is 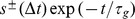. The process is illustrated for positive spike timing in Figure 6. Synaptic weights are updated according to 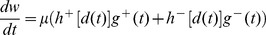
(8)


where 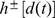 are functions of the (possibly changing) dopamine level 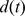, and 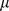 is a learning rate.

We now put 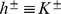, where 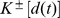 are the plasticity factors given by (6), but allowing time-dependent dopamine 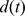. Then, using the first relation in (6), the learning rule for a single spike pair becomes 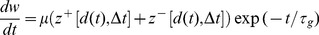
(9)


Here, the factor 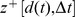 is given by the same functional form as (4) but now has a time-dependence with dynamically changing dopamine. The effects of multiple spike pairs are assumed to add linearly.

The complete STDE learning rule for a single synapse is thus given by Equation 9, which uses the STDP kernel 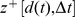 from Equation 4 defined by mixing the extreme STDP kernels in Equation 3 with the mixing function in Equation 5. The dynamic dopamine level 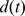 is specified by the modeller: for our simulated operant conditioning experiment we specify the within- and between-trial changes in dopamine below.

The choice of learning rule for STDE was dictated by the constraint that STDE reduces to STDP for constant levels of dopamine. Thus, integrating (9) gives the total change in weight due to the spike pair and, for constant dopamine, this is equal to the change for STDP in Equation (7) (up to the time constant 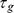, which may be absorbed into 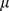).

### The Spiking MSN Model

The spiking model MSN is based on that in [45]. Essentially, this is an Izhikevich model [106] of a MSN, with the addition of direct dopaminergic modulation of both synaptically induced and intrinsic membrane currents. In the biophysical form of the Izhikevich model neuron [107], 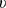 is the membrane potential and the “recovery variable” 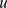 is the contribution of the neuron class’s dominant ion channel: 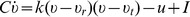
(10)

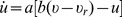
(11)


with reset condition

if 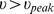 then 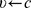, 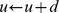


where, in the equation for the membrane potential (10), 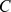 is capacitance, 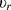 and 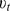 are the resting and threshold potentials, 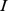 is the current due to synaptic input, and 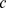 is the reset potential. Parameter 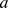 is a time constant governing the time scale of the dominant ion channel. Parameters 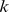 and 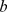 are arbitrary scaling constants, with the sign of 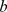 controlling whether the neuron is an integrator (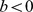) or a resonator (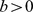). Parameter 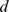 describes the after spike reset of recovery variable 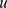, and can be tuned to modify the rate of spiking output.

The MSN model’s parameter values and their sources are given in Table 2. In [45] we showed how this model can capture key dynamical phenomena of the MSN the slow-rise to first spike following current injection; paired-pulse facilitation lasting hundreds of milliseconds; and bimodal membrane behaviour emulating up- and down-state activity under anaesthesia and in stimulated slice preparations.

**Table 2:** Intrinsic and synaptic parameters for the medium spiny neuron model.

Parameter	Value	Source
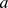	0.01	[107],[114]
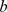	−20	[107]
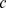	−55 mV	[107]
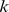	1	[107]
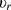	−80 mV	[107]
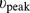	40 mV	[107]
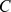	15.2 pF	[45]
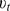	−29.7 mV	[45]
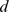	91	[45]
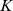	0.0289	[45]
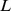	0.331	[45]
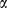	0.032	[45]
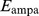 , 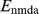	0 mV	[105]
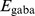	−60 mV	[105]
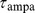	6 ms	[105]
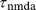	160 ms	[105]
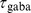	4 ms	[105]
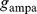	0.46 nS	rescaled from [45]
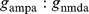	2	[105]
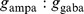	1.4	[105]
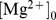	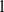 mM	[108]
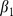	6.3	[45]
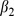	0.215	[45]

Synaptic input comprises the source of current 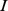 in Equation 10: 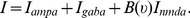
(12)


where 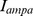, 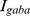, 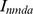 are current input from AMPA, GABA, and NMDA receptors, respectively, and 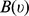 is a term that models the voltage-dependent magnesium plug in the NMDA receptors. Each synaptic input type 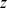 (where 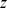 is one of ampa, nmda, gaba) is modelled by 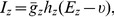
(13)


where 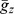 is the maximum conductance and 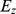 is the reversal potential. We use the standard single-exponential model of postsynaptic currents 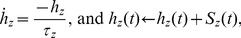
(14)


where 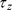 is the appropriate synaptic time constant, and 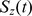 is the number of pre-synaptic spikes arriving at all the neuron’s receptors of type 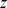 at time 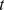.

The term 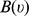 in Equation (12) is given by [108]

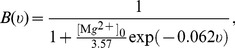
(15)


where 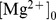 is the equilibrium concentration of magnesium ions.

Synaptic conductances were initialised with Gaussian noise so that they have a coefficient of variation of 0.1. Any synapses with negative conductance as a result of this initialisation was set to 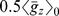. There was a ceiling on the synaptic conductance of 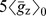.

### Dopaminergic Modulation of Ion Channels and Input

The following models of dopamine modulation are detailed in [45]. Let 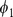 and 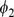 be the proportion of activated D1 and D2 receptors. For activation of D1 receptors we used the linear mappings: 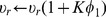
(16)


and 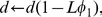
(17)


which respectively model the D1-receptor mediated enhancement of the inward-rectifying potassium current (KIR) (16) and enhancement of the L-type Ca^2^+ current (17).

For activation of D2 receptors we used the linear mapping: 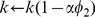
(18)


which models the the small inhibitory effect on the slow A-type potassium current, increasing the neuron’s rheobase current [105].

We add D1 receptor modulation of NMDA receptor evoked EPSPs by 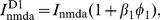
(19)


and we add D2 receptor modulation of AMPA receptor evoked EPSPs by 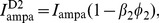
(20)


where 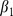 and 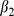 are scaling coefficients determining the relationship between dopamine receptor occupancy and the effect magnitude.

The dopamine dependent factors 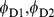 used in the dopamine-modulated neuron model are related to dopamine level 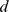 by 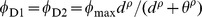, where 
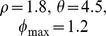
. This ensured that, for most of the phasic dopamine signal, 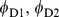 are both almost 1.

### Input Spike Trains

The neuron incorporated 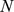 excitatory and 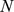 inhibitory (GABAergic) synapses, with 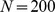. Each excitatory synapse contained a model of NMDA and AMPA receptors, as described above. Every synapse received a Poisson train of spikes at some specified firing rate. For the main experiments with operant learning, the GABAergic synapses received background input at three spikes/s; for the replication of the STDP protocols, they received no input. The firing rates of the excitatory synapses are detailed below.

### Simulating the Behavioural Experiment

#### Single trials

The structure of a single trial during learning is shown in components of Figure 6. The duration of each trial (time between consecutive bouts of high salience) was 2.4 s. The duration of the salience 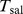, was 0.4 s. Of the 200 excitatory synapses to the model neuron, 50 were chosen at each trial to receive highly salient input; we refer to this as subset 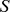, and its set complement with respect to all the synaptic inputs, 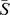. The background firing rate 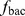 was three spikes/s and that during the salient period, 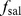 was 25 spikes/s. These are plausible figures for background cortical rates [109] and activity during motor behaviour [110]. If data were available, then more advanced models of the cortical input could take into account distributions of rates over the cortical inputs and their temporal dynamics over a single trial [111].

The range of dopamine level 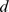 occupies a scale 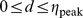, where 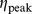 is the maximum amplitude of the phasic burst. The scale here is arbitrary and simply fixes a corresponding range of parameters that describe the effects of dopamine via the mixing function defined above. Thus, 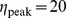 and the tonic dopamine level was 3. The phasic dopamine signal on a particular trial had an onset amplitude sampled from a Gaussian with a mean of the current value of 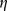 and standard deviation of 0.55, and decayed with a time constant of 20 ms [112] after onset. The time of delivery after the salient period, 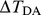, was 150 ms. The spike pair functions derived from the STDP, 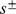, were assigned a plausible time constant of 20 ms, based on similar, STDP counterparts [113]. The eligibility time constant 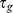 was 300 ms and was chosen so that the eligibility signal can interact substantially with phasic dopamine at the typical latencies encountered with this signal.

The conductances of all AMPA synapses were continuously updated over a trial using the STDE rules, with the set of 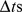 for each synapse defined by the times between the sets of pre-synaptic spike input and the set of postsynaptic spikes (when 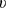 crosses 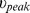).

#### Single experiment

Figure 1 shows the structure of a complete, multi-trial experiment. The number of trials in each of the epochs baseline, learning, intermission, extinction, post-extinction phases were 15, 40, 30, 40, and 30, respectively. During the learning and extinction phases, the same subset 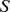 was chosen on every trial, representing the action being reinforced or extinguished. Outside these phases, the subset 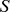 was randomly chosen on each trial.

Phasic dopamine decayed over the entire experiment with a time constant 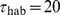
s, so that its amplitude 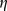, was just less than 1% of its maximal value 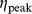 at the end of the learning epoch.

### Renewal and Reacquisition Tests

#### Data interpretation

Nakajima and colleagues [49] reported response rate data for learning, extinction, and renewal in the sequences 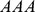, 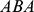, and 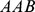 in their Figure 3. To control for the effect of changing the context alone on renewal performance, we wanted to compare performance in that context both before and after extinction (the latter being the “renewal” test). We thus interpreted the response performance during the first block of extinction as the before-point, and plot those data as “acquis.” in Figure 8A (note that Nakajima and colleagues used two sequences with the same renewal context 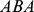 and 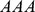
—the performance in the first block of extinction was similar for both, so we plot the average of the two). The data plotted as “renewal” in Figure 8A are taken from the first block of renewal trials.

For a further control, and for consistency with the experimental dataset on reacquisition (see below) we also required data for the sequence 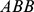. While, there is no such data point in [49], we assume renewal in 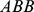 will be similar to that in 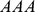, as reported by Crombag and colleagues [48].

To summarise the experimental data on 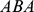 and 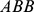 reacquisition performance from [50] we performed a linear regression on the data in their Figure 2 for each reacquisition context 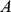 or 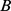 (which was originally reported for ten time-points); the plotted symbols in Figure 8B are the endpoints of those regressions at the two extreme times.

#### Fitting rate-coded units

We took the set of learnt synaptic weights for D1 and for D2-MSNs at the specified point in the simulated renewal or reacquisition experiments, and constructed equivalent rate-coded models that matched their input-output firing rate curves. First we found the input-output function for each spiking MSN model using those weights, and converted the function into its normalised rate-coded equivalent. For outputs, we assumed that 40 spikes/s corresponded to a normalized output rate of 1. For inputs, we assumed that a normalized value of 1 corresponding to all 50 highly salient inputs (in set 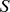) firing at 40 spikes/s (note that the rate-coded model input is a scalar, collapsing across all afferents to the spiking model). To then obtain the best fit with the target spiking input-output function, we varied the scalar input weight (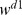 or 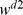) and the threshold 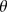. Fits were determined using a simple least squares method in MATLAB over a range of input values across the whole, normalized range. For examples of fitted models see Figure S2 and Text S2.

### Exhaustive Search for Plasticity Coefficients

Details are given here of the search for plasticity coefficients 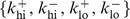 that give rise to MSN response profiles of the form in Figure 1. The 4D space of coefficients was divided into a regular rectangular lattice defined by the intersection of five regularly spaced points along each of the axes (giving 625 points). This was augmented by a point corresponding to the coefficients used in the data-constrained experiments reported in Figure 7. At each lattice point, three experiments were run using the experiment defined by Figure 1, but the numbers of trials in some epochs were reduced to expedite computation. Thus, for D1-MSNs, the number of trials in each epoch (baseline, learning, intermission, extinction, post-extinction) was reduced to 15, 30, 30, 20, 15, respectively, and for D2-MSNs, to 15, 40, 30, 20, 15.

Initially, the lattice was rather coarse grained with a liberal range of values; we were keen not to exclude any non-intuitive combinations of coefficient values. For D1 MSNs, the lattice was defined by drawing the coefficients from the five equi-spaced values across the following intervals: 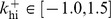, 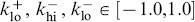. For D2 MSNs the intervals were 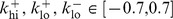. However, a second search was then conducted using a smaller lattice, whose domain was restricted by the more successful experiments from the first pass. For D1 MSNs this was given by 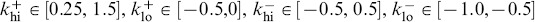, and for D2 MSNs by 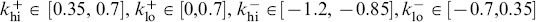


For each group of three experiments at each lattice point, the spike counts at each trial 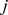 were averaged over this group, and across a window of three trials. These smoothed, ensemble-mean spike counts 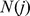 were then characterised with a feature-based metric in terms of their match to the target profiles in Figure 1. This metric was used as a guide for selecting MSNs with well-matched profiles, and fit to the target was ultimately corroborated by visual inspection (any feature-based method is only as good as the quality of the features it uses).

### Validating the Model against the Shen and ColleaguesData

We simulated the cortico-striatal plasticity induction protocols described in Shen and colleagues [17] using the spiking MSN model with a single AMPA synapse. They used a theta-burst protocol, with an asymmetric design for the positive (pre-post) and negative (post-pre) spike timing tests. For the pre-post test, each burst was three pre-synaptically induced EPSPs spaced by 20 ms, each EPSP followed by a fictive postsynaptic spike after 5 ms. For the post-pre test, each burst was three fictive postsynaptic spikes spaced by 20 ms, the last spike followed by a pre-synaptically induced EPSP after 10 ms. For both tests, the bursts were presented in blocks of 5 at 5 Hz (that is, the first event of a burst occured every 200 ms), and ten blocks were presented at 0.1 Hz (i.e., every 10 s).

To simulate this protocol we used a single synaptic input obeying the STDE rules to which we applied afferent spikes, and generated artificial postsynaptic spikes with the correct timing relations. The only difference was the extended period of time between blocks of stimuli was reduced to 2 s to avoid unnecessarily large simulation times (the neural membrane had returned to rest over this time, and all time constants in the model are substantially shorter than 2 s). Ten blocks of stimuli with potential plasticity were used, sandwiched between blocks with no plasticity (learning rate of zero), which served to allow measurement of mean EPSPs before and after learning. In line with the protocol of Shen and colleagues [17], the membrane potential was set to an initial holding value of −70 mV (by current injection). At no time were any spontaneous action potentials generated so that all spike pairs were synthetically created by the spike-pair timing protocol.

## Supporting Information

Figure S1 **To accompany Text S1.** D2 MSN activity is necessary for ideal action selection. (A) Dependence of basal ganglia model selection performance on the weight of cortical input to the action-coding D2 MSN population. We input a single pair of high-salience inputs to the model (0.7 to channel 1, and 0.6 to channel 2). For a range of cortical input weights to the D2 MSN population in channel 1, we plot the resulting equilibrium values of the basal ganglia output in channels 1 and 2, and their respective inputs from the STN, D1 MSN, and GPe populations. We see that there exists an intermediate range of cortical input weights to D2 MSNs for which successful selection of the highest salience input to channel 1 is achieved; otherwise either selection of both channels (for lower weights) or neither channel (for higher weights) occurs. (B) Examples of selecting both, one, and neither channel in the basal ganglia output with increasing cortical input weight to D2 MSNs. The input is shown in the top panel, and the output in the subsequent three panels; signals pertaining to channels 1 and 2 are shown by dashed and solid lines, respectively.
(TIFF)

Figure S2 **To accompany Text S2.** Exercising the trained MSNs in the model of basal ganglia. (A) and (B) show the process of finding rate coded MSNs equivalent to their spiking counterparts at the end of the intermission epoch. (A) Result of fitting spiking MSN responses to piecewise linear functions. The symbols show the normalised input/output firing rates for the spiking MSNs (triangles/circles are for D1 and D2-MSN, respectively). The lines show best piecewise linear fits (solid and dashed are D1 and D2, respectively). (B) The responses of D1 and D2-MSNs of the control channel in the rate coded model. (C) Show the outcomes in a two-channel competition in the model basal ganglia with bubble plots of the form used in Figure 3 (main text). The left, middle, and right hand panels show, respectively, the baseline response, trained MSNs at the end of intermission, and the end of extinction.
(TIFF)

Text S1 **Low-level D2 MSN activity is necessary for ideal action selection.**
(PDF)

Text S2 **Validating the model: MSN functionality is consistent with original target behaviour.**
(PDF)

## Abbreviations

LTD: long-term depression
LTP: long-term potentiation
MSN: medium spiny neuron
STDP: spike-timing dependent plasticity
TD: temporal difference
