# Correction: A New Framework for Cortico-Striatal Plasticity: Behavioural Theory Meets In Vitro Data at the Reinforcement-Action Interface

**DOI:** 10.1371/journal.pbio.1002099

**Published:** 2015-03-05

**Authors:** The PLOS Biology Staff

It has come to the authors’ attention that there are errors in Fig. 3 and in Fig. 8. In Fig. 3A and B, the dotted lines in the D2 response ‘heat maps’ were inadvertently missing and have now been added. Additionally, the x-axis gridlines have been removed. In the lower half of Fig. 8D, the color of the S_B_ line has been adjusted from black to blue, and the line for S_A_ under reacquisition has been adjusted from dark blue to light blue. The corrected versions are provided below.

**Fig 3:**
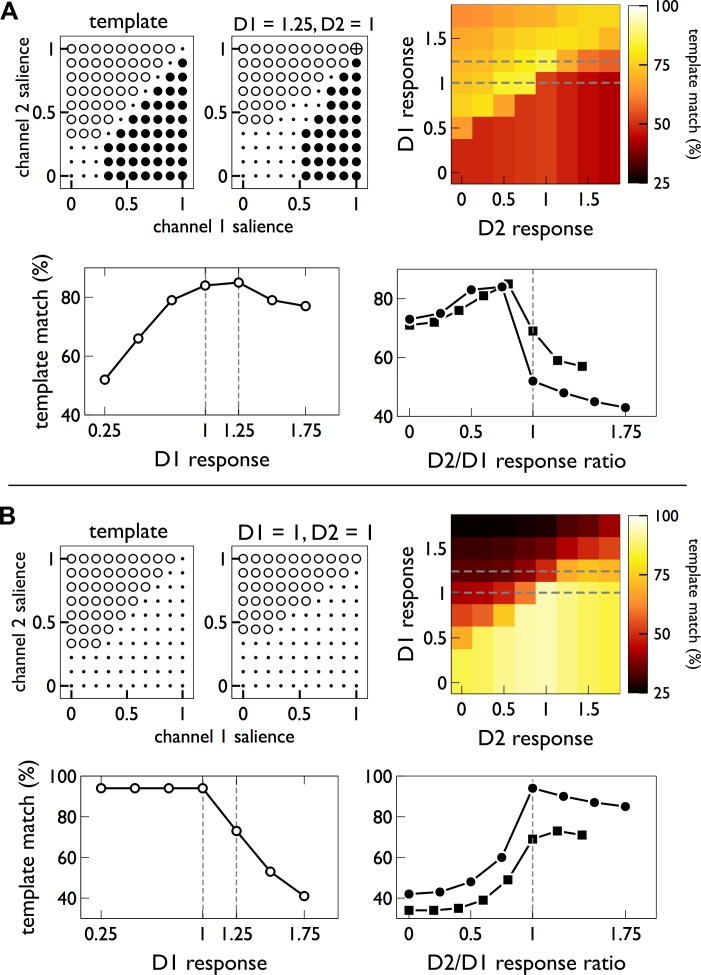
Linking action selection in basal ganglia to MSN responses. In all plots, neural “responsiveness” is the ratio of the population′s input value to output response; we abbreviate to “response” in axis labelling for brevity. (A and B) relate to action learning and extinction, respectively. The pairs of “bubble plots” in the top left of each panel show (i) an idealised selection template for a two-channel competition (left plot in each pair), with the key action on channel 1 and the control action on channel 2; and (ii) the best match to that template (at the D1 and D2 responsiveness noted above the plot). In each bubble plot, open symbols show an outcome of channel 2 selected, closed symbols show channel 1 selected, dots are no selection, and the crossed-circle shows both channels selected. The 2D colour plots (“heat maps”) show the template match for each D1/D2 responsiveness pair. The pairs of line plots show details of the corresponding colour map. The left hand line plots (open symbols) show the maximum template match for a given D1-MSN responsiveness; results at 1 and 1.25 are highlighted by the dashed lines. The right hand line plots (closed symbols) show cross sections through the 2D heat map (indicated by dashed grey lines therein) at D1 responsiveness of 1 (circles) and 1.25 (squares).

**Fig 8:**
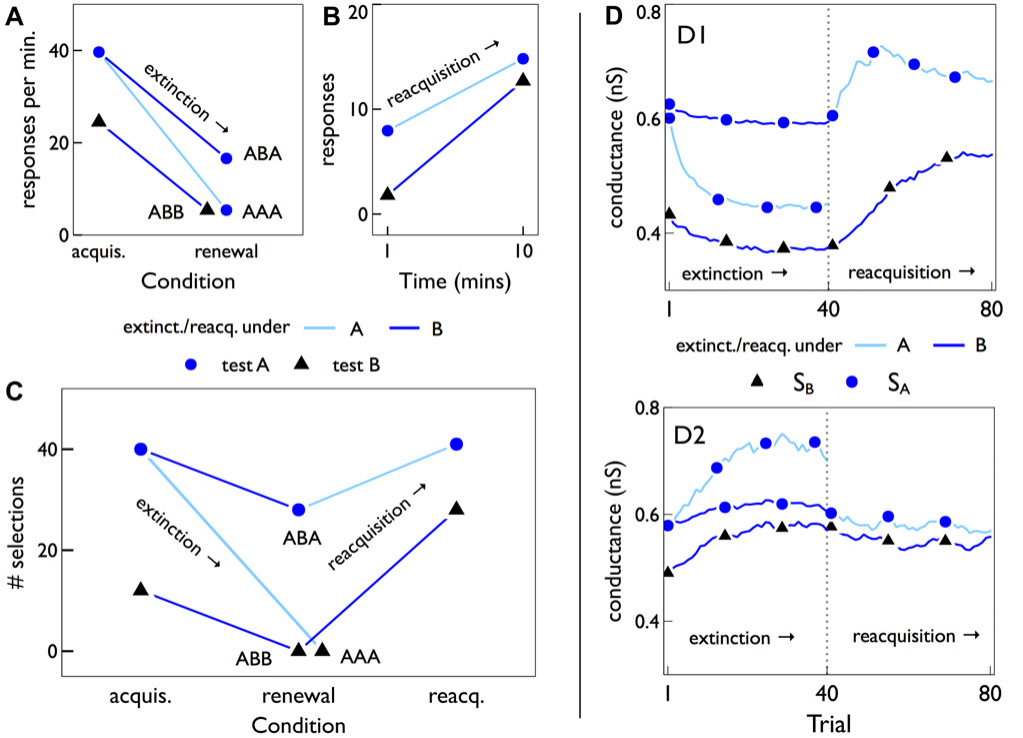
Extinction, renewal, and reacquisition. (A) Summary of relevant data from Fig. 3 in [49], (see Methods for interpretation of that data) showing renewal effects after sequence ABA, but not after control sequences AAA and ABB. The points labelled “acquis.” are the performance before extinction in the same context as the renewal test, giving a baseline for the performance change caused only by any switch in context after extinction. In all of (A–C), the blue/black symbols correspond to testing with contexts *A*/*B*. (B) Summary of relevant data from [50] (see Fig. 2 therein), showing reacquisition of responding in two contexts *A*,*B*, after original acquisition in *A* and extinction in *B*. The symbols show endpoints of linear regressions through the original data, which include outcomes at several intermediate time points. (C) Behavioural responses of the basal ganglia model with MSNs initially trained with context *A*. The acquisition (“acquis.”) is tested near the end of the intermission period for two contexts, *A*,*B*, derived using different strong-afferent synaptic sets *S*_*A*_,*S*_*B*_ (see text for details). The renewal is tested at the end of 40 trials of extinction under both contexts, leading to the renewal sequences *ABA*, *ABB*, and *AAA*. Reacquisition is measured after 40 learning trials, under each context. (D) Shows (for both D1- and D2-MSNs) the mean AMPA conductance of synaptic sets *S*_*A*_,*S*_*B*_ against trial number, during extinction (trials 1–40), and reacquisition (trials 41–80) under the behavioural protocols in (C). Trials are numbered from trial 80 near the end of the intermission period in the simulated experiment (Fig. 7). The trajectory for *S*_*A*_ under extinction with *A* (pale blue line, dark blue symbols) is identical to the extinction shown in Fig. 7.
