# Supplementary figures and images for: A New Framework for Cortico-Striatal Plasticity: Behavioural Theory Meets In Vitro Data at the Reinforcement-Action Interface
Source: PLoS Biol. 2015 Jan 6;13(1):e1002034. doi: 10.1371/journal.pbio.1002034 (PMC4285402; doi:10.1371/journal.pbio.1002034)

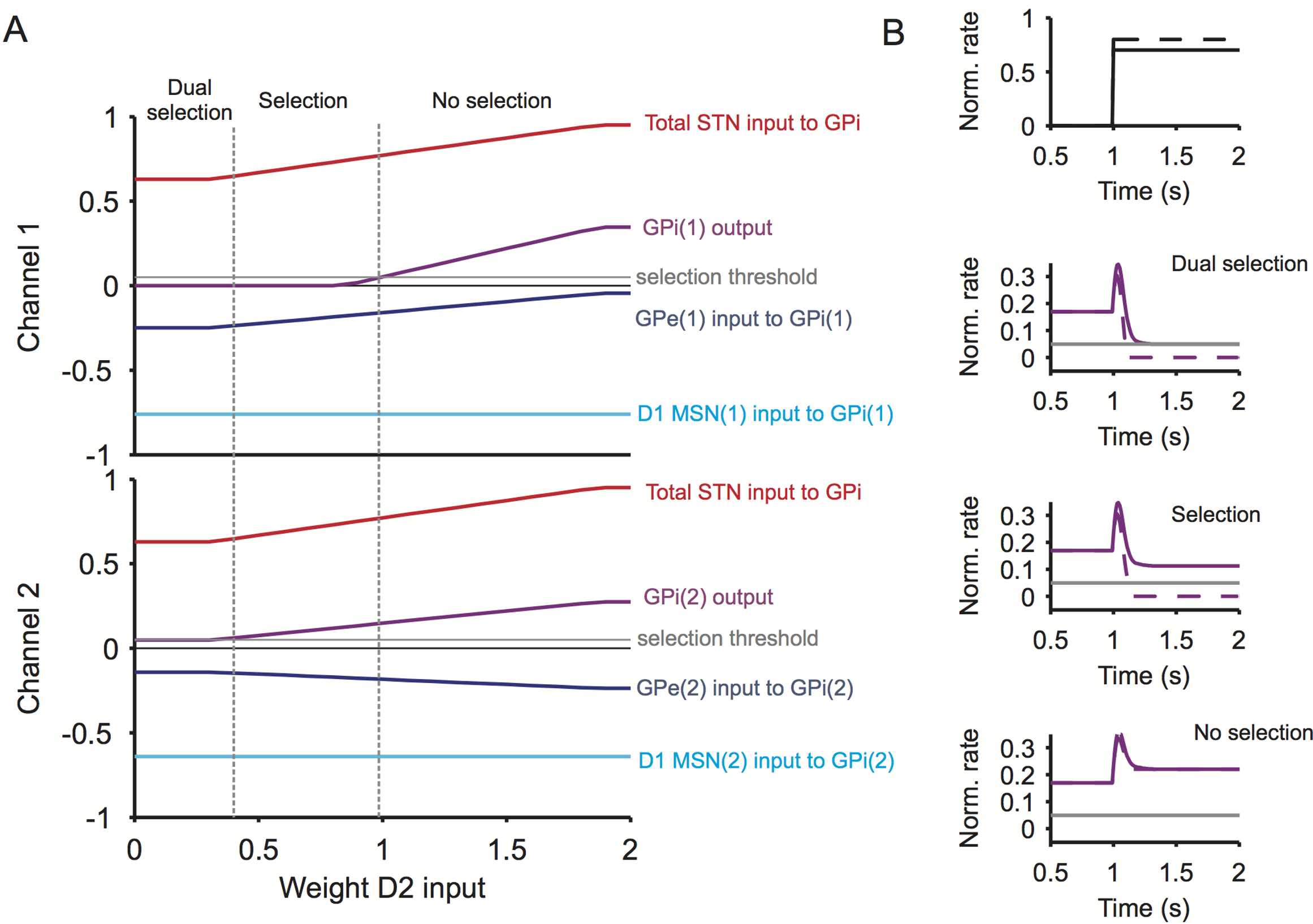

Supplement: Figure S1 — To accompany Text S1. D2 MSN activity is necessary for ideal action selection. (A) Dependence of basal ganglia model selection performance on the weight of cortical input to the action-coding D2 MSN population. We input a single pair of high-salience inputs to the model (0.7 to channel 1, and 0.6 to channel 2). For a range of cortical input weights to the D2 MSN population in channel 1, we plot the resulting equilibrium values of the basal ganglia output in channels 1 and 2, and their respective inputs from the STN, D1 MSN, and GPe populations. We see that there exists an intermediate range of cortical input weights to D2 MSNs for which successful selection of the highest salience input to channel 1 is achieved; otherwise either selection of both channels (for lower weights) or neither channel (for higher weights) occurs. (B) Examples of selecting both, one, and neither channel in the basal ganglia output with increasing cortical input weight to D2 MSNs. The input is shown in the top panel, and the output in the subsequent three panels; signals pertaining to channels 1 and 2 are shown by dashed and solid lines, respectively. (TIFF) [file pbio.1002034.s001.tiff]
