# Supplementary material for: A New Framework for Cortico-Striatal Plasticity: Behavioural Theory Meets In Vitro Data at the Reinforcement-Action Interface
Source: PLoS Biol. 2015 Jan 6;13(1):e1002034. doi: 10.1371/journal.pbio.1002034 (PMC4285402; doi:10.1371/journal.pbio.1002034)
Supplement: Text S1 — Low-level D2 MSN activity is necessary for ideal action selection. (PDF) [file pbio.1002034.s003.pdf]

Low-level D2 MSN activity is necessary for ideal action selection

A striking prediction of our basal ganglia network model is that successful selection of an action is best achieved when there is some activity in its coding D2 MSN population (see Figure 3, main text). As this prediction seems to contradict the basic select/suppress hypothesis for the pathways originating in D1 and D2 MSN populations, we here explain why the model makes this prediction.

To illustrate the underlying mechanisms, we simulated the basal ganglia network model with a pair of closely-matched inputs to channels 1 and 2 for a range of cortical input weights to channel 1's D2 MSN population. Figure S1A plots the resulting equilibrium values of activity in those two channels throughout the model as a function of the D2 MSN input weight. Figure S1A plots the resulting equilibrium values of activity in those two channels throughout the model as a function of the D2 MSN input weight. Figure S1B plots three example simulations showing the respective selection of both, one, and none of the channels with increasing D2 MSN input weight.

With no or low weighted input to the D2 MSN population, both channels are selected as both receive sufficient direct inhibition from their respective D1 MSN and GPe populations. With an intermediate D2 MSN weight only the most salient channel is selected: the decrease of GPe output by its D2 MSN input causes a rise in total STN output, which in turn drives a rise in GPi output on the less salient channel, but *not* the most salient channel. This is because the combined GPe and D1 MSN input to GPi on that channel is still sufficient to hold the population below its firing threshold. With a higher D2 MSN weight no channel is selected: the further decreased GPe output now means that its combination with the D1 MSN output is not sufficient to counteract the further increased total STN output.

We thus see that the D2 MSN output controls the balance of input to all GPi channels by controlling the activity in the STN-GPe feedback loop. Consequently, in a closely matched competition between action requests, some D2 MSN activity is necessary to ensure resolution of the competition in favour of a single action.
