# Supplementary material for: A New Framework for Cortico-Striatal Plasticity: Behavioural Theory Meets In Vitro Data at the Reinforcement-Action Interface
Source: PLoS Biol. 2015 Jan 6;13(1):e1002034. doi: 10.1371/journal.pbio.1002034 (PMC4285402; doi:10.1371/journal.pbio.1002034)
Supplement: Text S2 — Validating the model: MSN functionality is consistent with original target behaviour. (PDF) [file pbio.1002034.s004.pdf]

Validating the model: MSN functionality is consistent with original target behaviour

Here we 'close the loop' of the relationship between MSN responses and behaviour. That is, having established desired MSN response profiles from a behavioural analysis of basal ganglia output (Figure 1), we now seek to confirm that the MSNs derived from our learning rules give basal ganglia output of the required form.

Our goal here is to show that the responsiveness of the spiking D1 and D2 MSN models learnt through the dopamine-driven STDE rules can give rise to the required action selection and suppression at the end of leaning and extinction, respectively. To this end, we needed to test these models responsiveness in the rate-coded network model to allow a direct comparison to the original selection and suppression results (Figure 3).

We took the set of synaptic weights for D1 and for D2-MSNs at different epochs of the simulated operant task, and constructed equivalent rate-coded models that matched their input-output firing rate curves. Details of this procedure are contained in the Methods of the main paper text. There was a remarkably good fit of the MSN responses to piecewise linear functions of the form used in the rate-coded model (Figure S2A)

This is emphasised by comparing the fitted MSN functionality with the input/output characteristics of the control channel MSNs in Figure S2B. The main features of D1/D2-relative slopes,and intercepts with the x-axis, are similar in both cases. We should emphasise that the spiking MSNs are not *based* on their rate-coded counterparts but, rather, on a detailed compartmental model of the MSN [1, 2]. It is also important to note that the relative performance of D1-and D2- MSNs is reported using synaptic activity as input, and not injection current; results with the latter can be quite different [3, 1].

We then embedded these parameterised rate-coded D1 and D2 MSNs into the *experimental* channel of our basal ganglia network model and tested the model in the same two-channel competitions used in the main text (Figure 3) to establish the selection and suppression capability. Thus these network models incorporated the responsiveness of the D1 and D2 MSNs learnt through the dopamine-driven STDE plasticity rules. With these embedded the match with ideal selection templates was 76% post-learning (after intermission), and 87% for post-extinction (Figure S2C). This compares favourably with

the matches observed in Figure 3 of the main text. We conclude that the MSNs in the plasticity protocol do indeed give rise to expected basal ganglia behaviour.

References

1. Moyer JT, Wolf JA, Finkel LH (2007) Effects of dopaminergic modulation on the integrative properties of the ventral striatal medium spiny neuron. J Neurophysiol 98: 3731–3748.

2. Humphries M, Lepora N, Wood R, Gurney K (2009) Capturing dopaminergic modulation and bimodal membrane behaviour of striatal medium spiny neurons in accurate, reduced models. Frontiers in Computational Neuroscience 3.

3. Gertler TS, Chan CS, Surmeier DJ (2008) Dichotomous anatomical properties of adult striatal medium spiny neurons. The Journal of Neuroscience 28: 1081410824.
